# Exploring the mechanism of cordycepin combined with doxorubicin in treating glioblastoma based on network pharmacology and biological verification

**DOI:** 10.7717/peerj.12942

**Published:** 2022-02-15

**Authors:** Jing Chen, Yuan-Dong Zhuang, Qiang Zhang, Shuang Liu, Bing-Bo Zhuang, Chun-Hua Wang, Ri-Sheng Liang

**Affiliations:** 1 Fujian Medical University Union Hospital, Fuzhou, Fujian, China; 2 Fujian Medical University Union Hospital, Fuzhou, China

**Keywords:** Cordycepin, Doxorubicin, EMT, Network pharmacology, Drug combination, Glioblastoma

## Abstract

**Background:**

Glioblastoma is the most common and fatal primary malignant tumor in the central nervous system, and the prognosis is poor. Currently, there are no effective treatments for glioblastoma. Cordycepin is a natural active substance with significant anticancer activity and doxorubicin is a broad-spectrum anticancer drug. Cordycepin administered with doxorubicin is a potential drug combination for the treatment of glioblastoma. However, the mechanism of action for this drug combination has not yet been elucidated.

**Aim of the study:**

To explore the complex mechanism of cordycepin combined with doxorubicin against glioblastoma using network pharmacology and biological verification.

**Materials and methods:**

We used an MTT assay, colony formation assay, and scratch healing to detect the growth, proliferation, and migration of LN-229, U251 and T98G cells. Putative targets and the potential mechanism of action for the drug combination in glioblastoma were obtained through online databases, network construction, and enrichment analyses. We verified the expression of EMT-related genes and identified important therapeutic targets by western blot.

**Results:**

In this study, the combination of doxorubicin and cordycepin was found to significantly inhibit cell proliferation and migration and can induce apoptosis. These effects are better together than with either drug alone. The drug combination inhibited EMT by upregulating the expression of E-cadherin protein and downregulating the expression of N-cadherin, ZEB1, and Twist1 proteins. There were 71 potential targets for the drug combination in glioblastoma, and Kyoto Encyclopedia of Genes and Genome analysis suggested that the anticancer process may be mediated by proteoglycans in cancer, the tumor necrosis factor signaling pathway, microRNA in cancer, pathways in cancer, and other pathways. To study the molecular mechanism of anticancer activity, we detected the expression of target proteins with downregulated expression of NFKB1, MAPK8, MYC, and MMP-9 proteins and upregulated expression of cleaved caspase 3 that promoted the apoptosis of LN-229 cells.

**Conclusions:**

This study shows that the drug combination of doxorubicin and cordycepin effectively inhibits the growth and proliferation of LN-229 cells through multiple targets and multiple pathways, and the combination inhibits cell invasion and migration by regulating the EMT switch of tumor cells. Our findings provide new ideas about, and a theoretical basis for, the treatment of glioblastoma.

## Introduction

Glioblastoma is the most common malignant tumor of the central nervous system and is caused by the canceration of glial cells in the brain and spinal cord (Zhang, Niu & Zhao, 2021). It has the characteristics of a high degree of malignancy, strong aggressiveness, a high recurrence rate, and poor prognosis (Han et al., 2020). At present, either surgical resection and postoperative radiotherapy or combined chemoradiotherapy to improve the survival rate of patients have become the most important methods for treating glioblastoma (Jing, Zhang & Qin, 2021). The surgical plan for glioblastoma is relatively mature, but it also faces many difficulties, such as strong heterogeneity, different sensitivity to postoperative chemotherapy, low survival rate, and slow research and development of chemotherapy drugs for glioblastoma. These challenges also lead to limited clinical drug options, so a better treatment plan is urgently needed. In the fifth edition of the World Health Organization Classification of Tumors of the Central Nervous System (WHO CNS5) published in 2021, the LN-229 cell line used in this experiment belongs to glioblastoma (IDH wild type). It belongs to grade 4 glioblastoma in the classification (Louis et al., 2021).

Cordycepin, also known as 3′-deoxyadenosine, is an active component extracted from the fermentation broth of *Cordyceps militaris* (L.) Link and belongs to the purine alkaloid family (Tu, Wang & Xia, 2020). *Cordyceps militaris* (also known as *Cordyceps sinensis*) and cordyceps sinensis asexual-type stage are the same as the ergot fungal genus *Cordyceps sinensis*; according to the National Chinese Herbal Medicine record, “*Cordyceps sinensis* fruit body and insect body can be used as cordyceps sinensis medicine.” *C. militaris* is used in the development of anticancer drugs because of its antitumor effect (Tu, Wang & Xia, 2020; Zhou et al., 2018). Studies have shown that cordycepin mediates anticancer (Chen et al., 2017), antibacterial (Jang et al., 2015), anti-inflammatory (Li, Chen & Huang, 2019), and other biological activities by inhibiting mRNA polyadenylation and regulating various cellular processes (Bandak et al., 2017; Gao et al., 2021). Cordycepin is natural, safe, and highly active. Due to its various biological activities, it can be used in combination with chemotherapy drugs to reduce the toxic side effects of chemotherapy drugs. Cordycepin has an inhibitory effect on the growth of human leukemia cells (Liao et al., 2015; Park et al., 2017), breast cancer cells (Fan, Zhu & Zhao, 2019), liver cancer cells (Guo et al., 2019), cervical cancer (HeLa) cells (Tania et al., 2019), glioblastoma (U251) cells (Chaicharoenaudomrung, Jaroonwitchawan & Noisa, 2017), and other tumor cells, so it has a wide range of medical value. Studies have shown that cordycepin can inhibit the proliferation of brain cancer in SH-SY5Y cells and U251 cells, and it can induce the apoptosis of brain cancer cells in collaboration with chloroquine (Chaicharoenaudomrung, Jaroonwitchawan & Noisa, 2017). Doxorubicin is a widely used chemotherapy drug for acute leukemia, malignant lymphoma, breast cancer, ovarian cancer, neuroblastoma, and other cancers. Doxorubicin has been very effective against different strains of glioblastoma in experiments, but it cannot pass through the blood–brain barrier. After intravenous injection of doxorubicin, the blood concentrations required to kill cells in brain tumor tissues are difficult to achieve (Deng, Mo & Luo, 2011); at the same time, doxorubicin has strong toxic side effects and drug resistance, so its use has certain limitations. Research by Yuan (2017) suggests that cordycepin can improve brain injury caused by the destruction of the blood–brain barrier, but whether cordycepin can pass through the blood–brain barrier requires more study. Zhang et al. (2021) showed that cordycepin combined with doxorubicin could inhibit the migration of ACHN cells in renal cancer and significantly promote the expression of apoptotic proteins.

Currently, few systematic reports describe the mechanism of action for cordycepin combined with doxorubicin to treat glioblastoma. Network pharmacology is now widely used to discover the basis of pharmacodynamic substances and explore their molecular mechanisms of action. This research combines the biological verification and network pharmacology of cordycepin, doxorubicin, and glioblastoma targets; an analysis of the target protein interactions, Gene Ontology (GO) function analysis and Kyoto Encyclopedia of Genes and Genomes (KEGG) target analysis. The larger degree of targets in the protein–protein interaction (PPI) network, as detected by Western blot, to clarify the potential mechanisms and resistance potential for cordycepin combined with doxorubicin in glioblastoma.

## Materials and Methods

### Reagents, antibodies, and chemicals

Fetal bovine serum, RPMI 1640, DMEM, MEM culture medium and penicillin and streptomycin were purchased from Gibco (Grand Island, NE, USA). Cordycepin, doxorubicin and MTT were purchased from Sigma (Ronkonkoma, NY, USA). Trypsin was purchased from Beijing Solaibao Technology Co., Ltd.. Dimethyl sulfoxide was purchased from Sinopill Chemical Reagents Co., Ltd.. Primary antibodies against GAPDH, E-cadherin, N-cadherin, Zinc finger E-box binding homeobox 1 (Zeb1), Twist1, NFKB, MAPK8, MMP-9, and cleaved caspase 3 were purchased from Cell Signaling Technology.

The cordycepin CAS number is 73-03-0, and its HPLC is >98%. The doxorubicin CAS number is 25316-40-9, and its purity is 98.0%–102.0%.

### Cell culture

Human glioblastoma cells (LN-229, U251, T98G) came from the American Type Culture Collection (Manassas, VA, USA). After resuscitation, LN-229, U251 and T98G cells were respectively cultured in an RPM-1640, DMEM and MEM medium containing 10% fetal bovine serum and 1% antibiotics (penicillin and streptomycin) in a constant-temperature incubator with a volume fraction of 5% CO_2_ at 37 °C. The cells were grown to 90% on the wall and were digested and subcultured with 0.25% trypsin. The logarithmic-phase cells were taken for experiments.

In this study, we dissolved cordycepin and doxorubicin in complete medium.

### Cell morphology

LN-229, U251 and T98G cells in the logarithmic growth phase were diluted to 1 × 10^4^ cells/mL in a cell suspension, and 1 mL was inoculated on a six-well culture plate and cultured in an incubator at 37 °C with 5% CO_2_. After the cells were fully expanded, a control group and experimental groups were formed. The control group had 2 mL of complete medium. The experimental groups contained 80 μmol/L of cordycepin and 1 μmol/L of doxorubicin separately and the same concentrations of each drug in combination. The solutions for culture had a final volume of 2 mL each and were maintained at 37 °C and 5% CO_2_ in an incubator for 48 h. Microscopic observation at ×200 magnification was used to record the treatment group effects on glioblastoma cells.

The survival rate analysis was calculated as follows: cell survival rate = [OD_test group_ − OD_zero hole_] ÷ [OD_control group_ − OD_zero hole_] × 100%, in which OD represents optical density.

### Cell viability assay

The experimental method refers to the method of Chen, Wang & Li (2012), Wang & Li (2013) and Syam et al. (2014) and is modified. LN-229, U251 and T98G cells in the logarithmic growth phase were selected and counted under a microscope; then, the cell concentration was diluted to 1 × 10^4^ cells/mL and seeded into 96-well plates, with 100 μL per well. After the cell adherent was fully expanded, the drug was administered by treatment group: cordycepin alone, doxorubicin alone, or the combined treatment. At the same time, the control group without medicine was established. Each group was placed into six parallel wells for culture and was incubated overnight in 5% CO_2_ at 37 °C for 48 h. After the culture, the culture medium was discarded, and 20 μL of MTT (5 mg/mL) was added to each well. The culture was continued for 4 h; then, the culture medium was discarded, and 150 μL of dimethyl sulfoxide was added to each well and mixed thoroughly until the purple brown precipitate was completely dissolved. The OD was measured for each well at a wavelength of 490 nm. Survival analysis was calculated as follows: cell survival rate = [OD_experiment group_ − OD_zero pore_] ÷ [OD_control group_ − OD_zero pore_] × 100%. We conducted at least three repeated biological experiments to ensure the accuracy of the results.

### Colony formation assay

We took logarithmic LN-229, U251 and T98G cells and the cell suspension was diluted to 1 × 10^3^ cells/mL and inoculated onto a six-well culture plate. The control group was a complete medium (2 mL); the experimental groups contained cordycepin (80 μM), doxorubicin (1 μM), or 80 μM of cordycepin combined with 1 μM of doxorubicin in culture to a final volume of 2 mL. Each group was added to three wells, and the culture medium was replaced every 3 days. If the number of cells observed in the colony was greater than 50, the clone had formed, and the culture could be terminated. The drug-containing culture medium was absorbed, discarded, and washed with phosphate-buffered saline (PBS) three times. We fixed the colonies with glutaraldehyde (6.0%v/v) and a sufficient amount of crystalline violet (0.1%) was added and stained at room temperature for 30 min; then, the crystalline violet was absorbed, phosphate-buffered saline was added and the solution was rinsed three times until no obvious purple background was present. We counted using a stereomicroscope. Counting was determined as follows: cloning formation rate = [clone number of experimental group ÷ clone number of control group] × 100%. We conducted at least three repeated biological experiments to ensure the accuracy of the results.

### Wound healing

The experimental method refers to the method of Franken et al. (2006) and is modified. Three horizontal lines were drawn on the back of the sterile six-well plate and passed through the holes. The distance between each horizontal line was 0.5–1 cm. LN-299 cells in the logarithmic growth period were taken and counted under the microscope. Each well was inoculated with 1 mL of diluted cells to 1 × 10^4^ cells/mL and placed in an incubator at 37 °C and 5% CO_2_ for additional culture. When the cell length in the hole reached approximately 80–90%, perpendicular to the horizontal line behind the hole, the six-well plate after the scratch was placed under a microscope and photographed, and the scratch width of the original cell was recorded as 0 h. A 10% serum culture medium was added to the control and the experimental groups (cordycepin 80 μM, doxorubicin 1 μM and the same doses of cordycepin + doxorubicin together) for incubation after 48-h cultivation. Wells were photographed under a microscope at 0 h, and a photographic record of cell scratch healing was obtained, with records of the scratch width of cells for 48 h, according to the following formula: cell migration rate = [(original scratch width − current scratch width) ÷ original scratch width] × 100%. We conducted at least three repeated biological experiments to ensure the accuracy of the results.

### Network pharmacological analysis

#### Target screening of cordycepin, doxorubicin and glioblastoma

Basic information about cordycepin and doxorubicin was searched through the PubChem database in NCBI. The targets of doxorubicin were collected from SuperPred, the SEA Search Sever, GeneCards and other databases, and results were screened for duplication. The targets of cordycepin were obtained from the SEA Search Sever, Bingding DB, GeneCards and other databases, and results were screened for duplication. The human gene names were interchanged in the Uniprot database. Cordycepin had 146 targets, and doxorubicin had 2,494 targets. The keyword “glioblastoma” was input into The Human Gene Database (GeneCards) to retrieve glioblastoma-related targets, and 4,159 targets were obtained; the human gene names were exchanged using the Uniprot database. The obtained targets of cordycepin, doxorubicin, and glioblastoma were intersected, and a Venn diagram was drawn to extract the common target genes.

#### GO enrichment and pathway analysis

GO biological function: The intersection of the three targets was imported into the DAVID database, and the species *Homo sapiens* was selected for GO enrichment analysis. We obtained the results of the biological process analysis. Then, we selected the biological processes for which *p* was <0.01 to draw a bar chart, and the biological processes related to apoptosis, proliferation and metastasis were screened out.

KEGG pathway analysis: The predicted intersection targets were imported into the DAVID database for KEGG pathway enrichment analysis, and the top 20 pathways with *p* values <0.01 were selected in order from smallest to largest *p* value. The top four pathways with the smallest *p* value were selected for a bubble diagram, and the network topology diagram of the disease–drug–target–signal pathway was constructed and visualized in Cytoscape 3.8.0 software.

#### Core target interaction network construction and analysis

The first four pathway-related targets with the smallest *p* value in the anti-glioblastoma process of cordycepin combined with doxorubicin were imported into the STRING platform; the species was set as *Homo sapiens*; the protein interaction relationship was retrieved; the lowest interaction threshold (medium confidence = 0.4) was set; and the other parameters were kept at the default values. The results were saved as a TSV file and imported into Cytoscape 3.8.0 software. Network Analyzer tool was used for network analysis, and topological parameters such as degree value (connectivity), betweenness centrality value (centrality) and closeness of each network node were calculated. Using the Cytoscape software, the size and color of the node were correlated with the degree, and the thickness of the edge was correlated with the combine score, to draw the PPI target of the protein interaction network. The derived image reflected the PPI network for the anti-glioblastoma target effect of cordycepin combined with doxorubicin.

#### Western blot assay

The logarithmic growth cells were pretreated with cordycepin, doxorubicin, or cordycepin + doxorubicin to detect the expression of glioblastoma invasion and metastasis-related proteins (*e.g*., E-cadherin, ZEB1) and the expression of related proteins with an equivalent degree value in the PPI network. The cell lysates were prepared using a RIPA lysis buffer containing a mixture of protease inhibitors. Cell lysates containing 50 μg of protein were isolated on a 10% Tris-HCl gel and transferred to a polyvinyl fluoroethylene membrane, which was then protein sealed with 5% skimmed milk in 1× Tris-buffered saline (TBS). The blots were then incubated overnight with a 1:1,000 dilution of the primary antibody. The membrane was washed with TBS-T and then incubated in TBS with a secondary antibody conjugated with horseradish peroxidase (1:5,000 dilution). Finally, the film was washed and the color was developed with an enhanced chemiluminescence substrate. Bands of protein were observed on a gel imager. Protein measurements were standardized to the GAPDH protein. The experiment was conducted independently at least three times. The expression of related proteins was analyzed by Image J software.

### Statistical analysis

Data were expressed as the mean ± standard deviation, and the data were analyzed with SPSS version 25.0 software. One-way analysis of variance was used to compare different treatment groups, and *p* < 0.05 and *p* < 0.01 indicated significant and extremely significant differences, respectively.

## Results

### Drug combination to inhibit the activity of LN-229 cells

**Figure 1:**
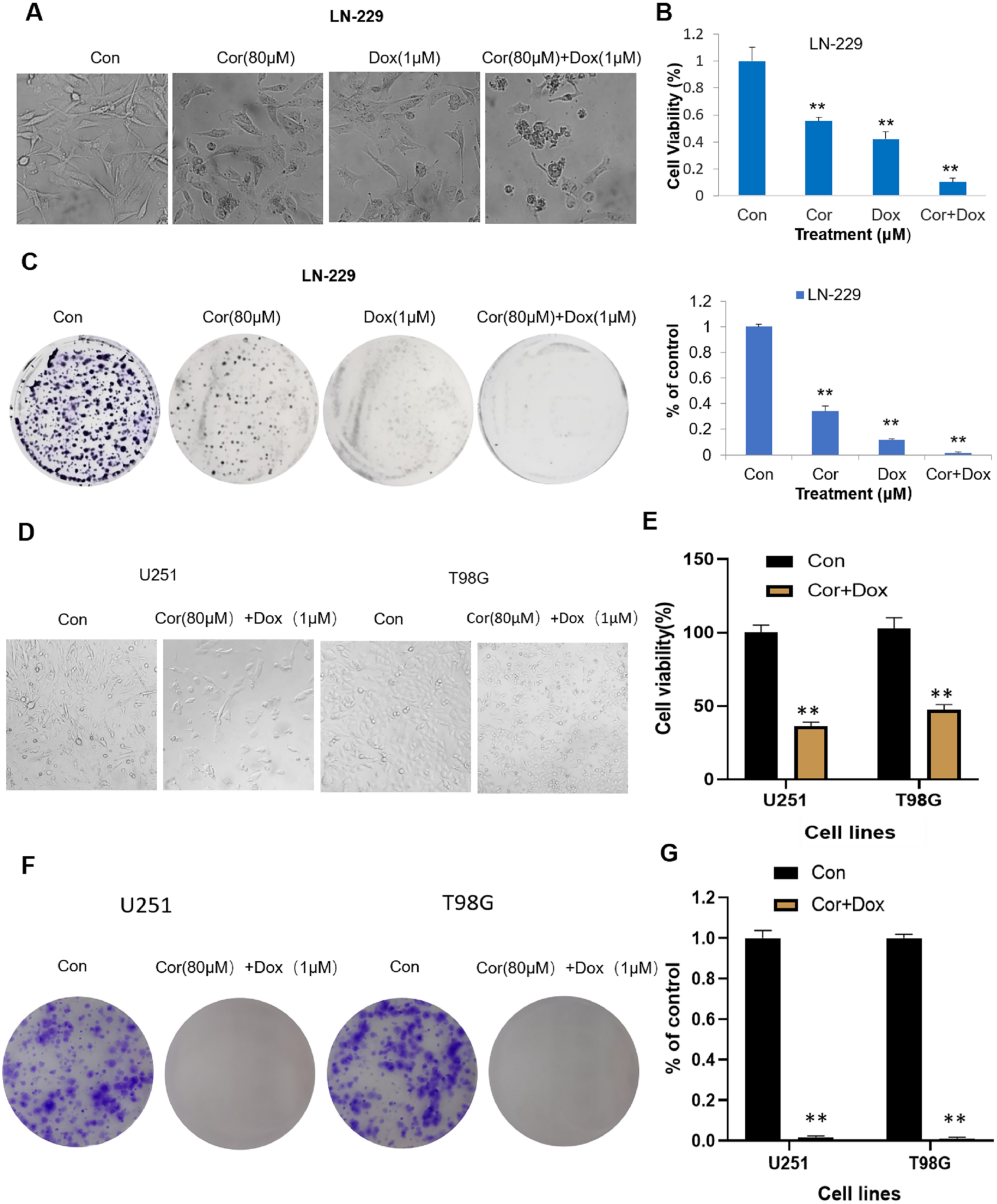
Cordycepin augments the effects of doxorubicin in glioblastoma cells. (A) LN-229 cells were treated with cordycepin (Cor, 80 μM), doxorubicin (Dox, 1 μM) or the combination (80 μM Cor + 1 μM Dox) for 48 h. (B) Cytotoxicity was detected by the MTT assay. (C) Clonogenic survival assay showing colony numbers of LN-229 cells after treatment with Cor (80 μM), Dox (1 μM), or the combination. Data are presented as the mean ± SD; *n* = 3; **p < 0.01 *vs* each group treated with only one drug alone. (D) U251 and T98G cells were treated with 80 μmol/L of cordycepin combined with 1 μmol/L of doxorubicin for 48 h. (E) The cell viability of both U251 and T98G cells treated with the combination drug decreased, which was significantly different from that of the control group. (F) A plate cloning experiment was performed on U251 and T98G cells, and the results showed that the combined drug could significantly inhibit the cloning ability of U251 and T98G cells. (G) Visualize the results of plate cloning experiments (*n* = 3 represents biological repetition).

Cordycepin combined with doxorubicin can inhibit the growth of glioblastoma LN-229 cells and induce their apoptosis. We first observed the morphological changes of LN-229 cells after 48 h of treatment with cordycepin, doxorubicin and cordycepin combined with doxorubicin. Figure 1A shows that, after 48 h, the growth rate of cells in the control group was faster and there were more adherent cells with a regular morphology and spindle shape. Compared with the number in the control group, the number of adherent cells treated with 80 μmol/L of cordycepin slightly decreased, and the cell morphology became slightly round. The number of adherent cells with doxorubicin decreased significantly, and there were some suspended cells. All the cells treated with the combination showed obvious morphological changes; the suspended cells increased and were broken and floating in fragments. Compared with the numbers with cordycepin or doxorubicin alone, the number of adherent cells was the least, the number of apoptotic and rounded cells was the most, and surrounding cells were more obvious in the combination group. These results indicate that cordycepin and doxorubicin alone were both toxic to LN-229 cells, cordycepin combined with doxorubicin on LN-229 cells was more effective obvious.

We then measured the cytotoxicity of the combination on LN-229 cells. MTT analysis (Fig. 1B) showed that cordycepin (80 μmol/L), doxorubicin (1 μmol/L), and the combined treatment gradually increased the cell inhibition rate; the cell survival rates for the treatments, respectively, were 58.97 ± 3.05%, 44.85 ± 5.75%, and 10.85 ± 3.04%. Overall, these results demonstrate that both cordycepin and doxorubicin could inhibit the viability of LN-229 cells and that cordycepin combined with doxorubicin was more inhibitory than that of the single treatment.

In order to better study the effect of cordycepin combination on glioma, we used two other cell lines (U251, T98G) for verification. The results of MTT analysis (Fig. 1D) showed that cordycepin combined with doxorubicin can significantly reduce the survival rate of U251 and T98G cells. The survival rates of U251 cells and T98G cells are respectively 36.33 ± 2.73% and 47.56 ± 3.51%. This shows that the combination of drugs can effectively inhibit the viability of U251 and T98G cells, and that cordycepin combined with doxorubicin has certain toxicity to cells of glioblastoma cell lines.

Figure 1C shows that compared with the control group, the number of clones formed by the cells in the cordycepin combined with doxorubicin group is extremely small. After calculation, Fig. 1G shows that the clone formation rate of U251 cells treated with the combination drug was 1.25 ± 0.02%, while the clone formation rate of T98G cells treated with the combination drug was 0.59 ± 0.01%, which was significantly different from that of the control group (*p* < 0.01). This shows that the test concentration is appropriate, and the combination of drugs can effectively inhibit the proliferation of U251 and T98G cells.

### Combined therapy inhibits the proliferation of glioblastoma cells

To study the effect of cordycepin combined with doxorubicin on the proliferation of LN-229 cells, a colony formation assay was carried out. The cell clone formation assay can be used to test the proliferative ability, invasiveness, sensitivity to killing factors, and other aspects of Fig. 1C shows that, compared with the number in the control group, the numbers of cell clones formed in the cordycepin group and the doxorubicin group were significantly reduced, and the number of cell clones formed in the combination group was very low. The ability to form clones successively decreased from the control group to the cordycepin group, the doxorubicin group, and the combined group. Figure 1D shows that the calculated clone formation rates of cordycepin, doxorubicin, and the combined group were, respectively, 34.18 ± 3.68%, 11.72 ± 0.94%, and 1.55 ± 0.83%, and the clone formation rates of the cordycepin and doxorubicin groups were, respectively, 22.05 and 7.56 times higher than that of the combined group (*p* < 0.01). These results indicate that the clone ability of LN-229 cells was significantly inhibited by cordycepin, doxorubicin, and the combination therapy, and the inhibition of the proliferation ability of LN-229 cells was stronger with the combination therapy than with the single therapy.

### Drug combination inhibits the invasion and migration of LN-229 cells by regulating epithelial mesenchymal transformation

**Figure 2:**
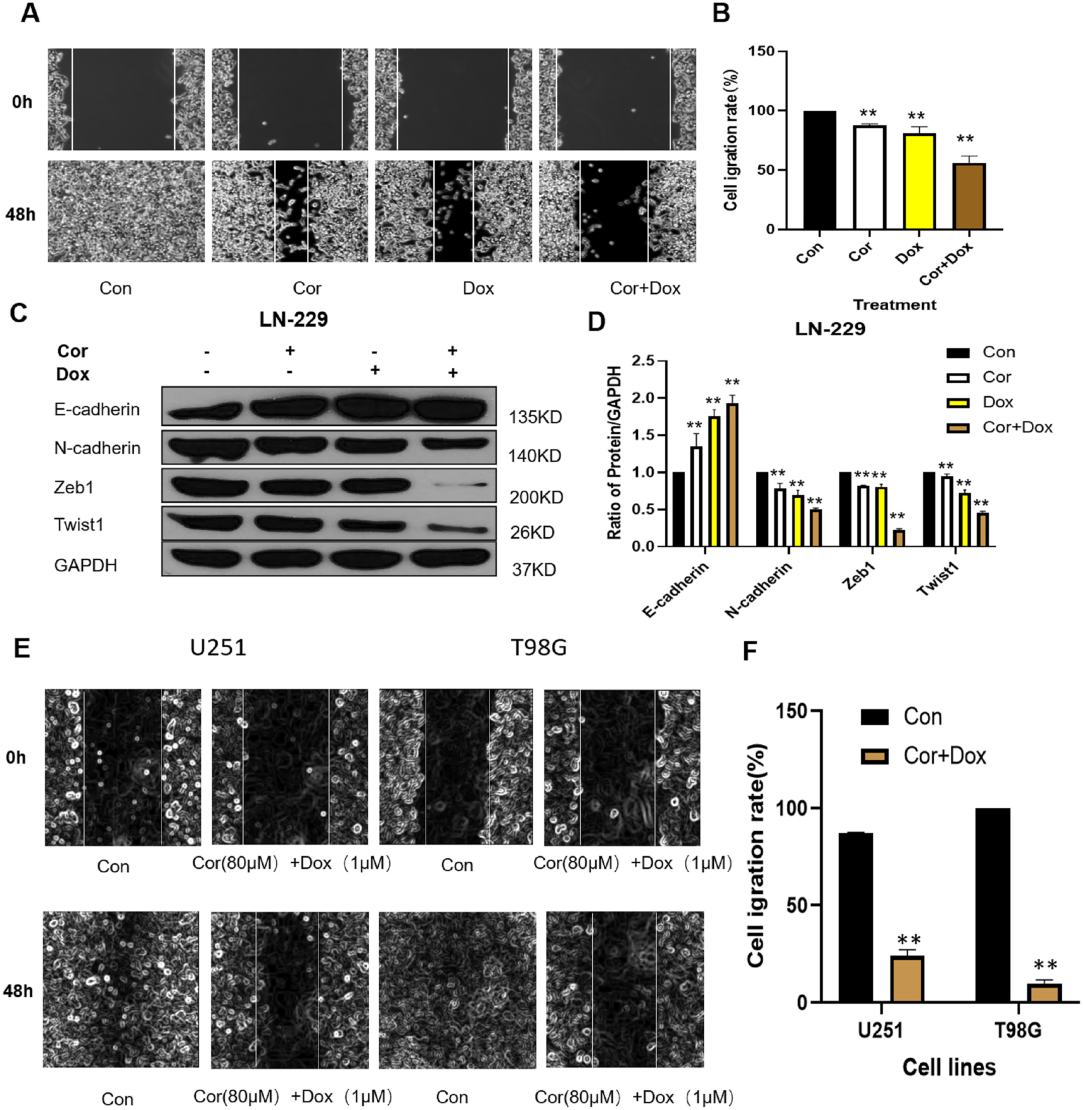
Effect of cordycepin combined with doxorubicin on cell migration. (A) A wound healing assay was conducted to evaluate the cellular migration of LN-229 cells. Representative images show the growth into wounded areas of LN-229 cells treated with cordycepin (Cor) and/or doxorubicin (Dox) at either 0 or 48 h after the scratch. (B) We used Image J software to quantify the cell scratch healing experiment. (C) Western blotting analysis shows the protein expression levels of EMT-related genes in LN-229 cells; all drug treatments alone or combined groups can upregulate the expression of E-cadherin protein and downregulate the expression of N-cadherin protein, Zeb1 and Twist1 protein. GAPDH was used as a reliable internal control. (E) A wound healing assay was conducted to evaluate the cellular migration of U251 and T98G cells. Representative images show the growth into wounded areas of U251 and T98G cells treated with cordycepin (Cor) and doxorubicin (Dox) at either 0 or 48 h after the scratch. (F) We used Image J software to quantify the cell scratch healing experiment. Data are presented as the mean ± SD; *n* = 3; **p < 0.01 *vs* the control group (*n* = 3 represents biological repetition).

Cell migration is one of the key steps in the invasion and metastasis of malignant tumors. The effect of the drug combination on the migration ability of LN-229 cells was detected by a cell scratch test, and the results are shown in Fig. 2. As seen in Fig. 2A, compared with 0 h, scratches healed after 48 h of cell culture in the control group and the experimental group. The healing abilities of different treatment groups were as follows: the control group more than the cordycepin group, doxorubicin group and combined group, indicating that cells migrated during the culture process. The healing ability of the control group was the strongest, indicating the greatest degree of cell migration, but the scratch healing of the combined group was the weakest. The combination of cordycepin and doxorubicin could effectively inhibit the movement and migration of LN-229 cells. Figure 2B shows that the calculated cell migration rates of the cordycepin group, doxorubicin group, and combined treatment group were, respectively, 87.95 ± 1.12%, 81.05 ± 5.52%, and 56.07 ± 5.85%, and extremely significant differences were documented between the combined group and the cordycepin group and the doxorubicin group (*p* < 0.01). Combination medication can effectively inhibit the migration ability of U251 and T98G cells (Fig. 2E). Figure 2F shows that the cell migration rate of cordycepin combined with doxorubicin on U251 and T98G cells was 23.97 ± 3.22%, 9.70 ± 2.04%, respectively.

To examine the molecular mechanisms underlying the epithelial mesenchymal transformation (EMT) process, we measured the expression of E-cadherin, N-cadherin, Zeb1, and Twist1 by western blot. The cordycepin group, doxorubicin group and combined group could regulate the EMT process by regulating EMT markers (*e.g*., N-cadherin, E-cadherin, ZEB1, Twist1) and could inhibit the expression of proteins related to tumor migration. EMT is a biological process in which epithelial cells transform into mesenchymal stem cells with characteristics driven by specific physiological or pathological factors (Yang et al., 2021)^.^ EMT plays an important role in the process of invasion and migration in glioblastoma. Figure 2C shows that, compared with the control group, the cordycepin group, doxorubicin group, and combined treatment group could upregulate the expression of E-cadherin protein and downregulate the expression of N-cadherin protein, ZEB1, and Twist1 protein. E-cadherin is a major mediator of cell adhesion and epithelial tissue integration, and it plays an important role in maintaining cell adhesion (Bremnes et al., 2002). The low expression or deletion of E-cadherin can inhibit intercellular adhesion, trigger tumor detachment from the primary site, and lead to invasion. The decreased expression level of E-cadherin protein was significantly correlated with the invasion and metastasis ability of tumor cells. N-cadherin is an important marker of EMT. ZEB1 is an important regulatory gene for EMT; it can inhibit the expression of E-cadherin and promote EMT and tumor invasion and metastasis (Zhao et al., 2018). The results of this study showed that, compared with expression in the control group, the expression of E-cadherin was upregulated in all the experimental groups. The expression of E-cadherin protein was the highest in the combined treatment group, whereas the expression of N-cadherin protein was low, thus reducing the invasion and metastasis of glioblastoma.

### Candidate targets of cordycepin, doxorubicin and glioblastoma

After human gene name replacement, we gained 146 and 2,494 potential targets of cordycepin and doxorubicin, respectively. The PubChem CID, molecular formula, and number of targets of cordycepin and doxorubicin collected from the PubChem compound database in NCBI are shown in Table 1.

**Table 1:** Main information of cordycepin and doxorubicin.

Compound	PubChem CID	Molecular formula	Target number
Cordycepin	6,303	C_10_H_13_N_5_O_3_	146
Doxorubicin	31,703	C_27_H_29_NO_11_	2,494

**Figure 3:**
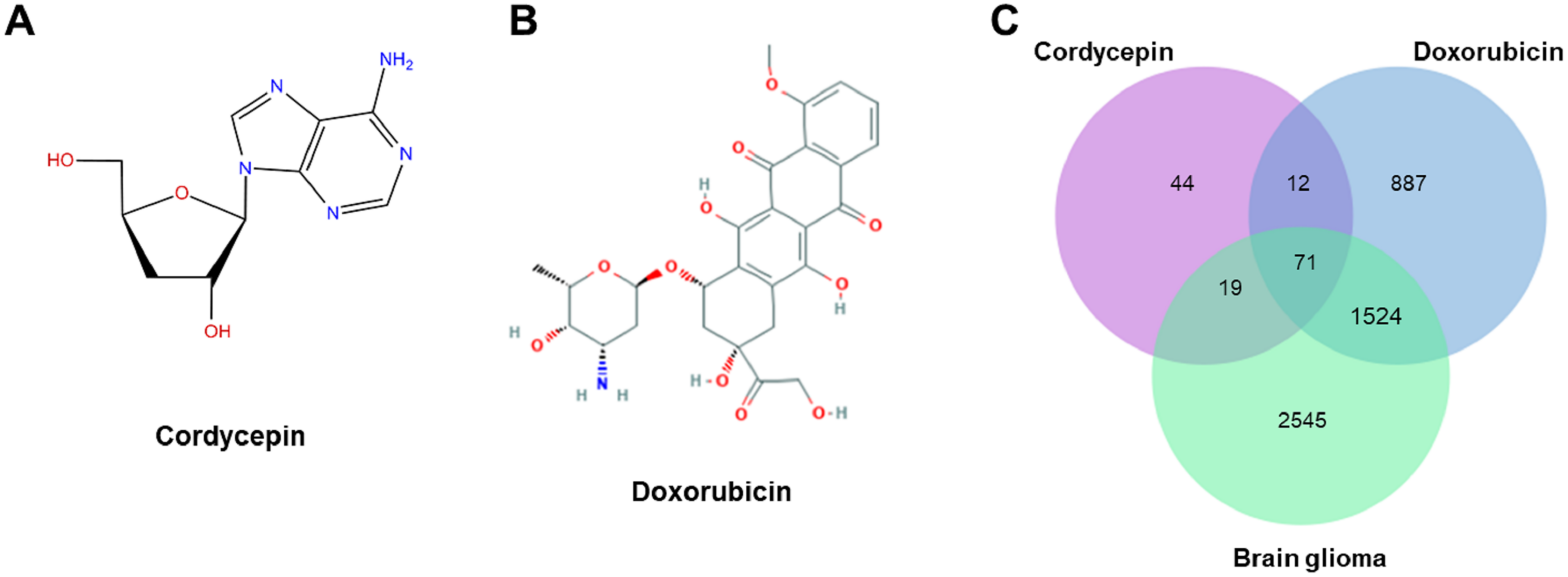
The structures of cordycepin and doxorubicin. (A) Schematic diagram of the structure of cordycepin. (B) Schematic diagram of the structure of doxorubicin. (C) Venn diagram of cordycepin, doxorubicin, and glioblastoma. We obtained 71 intersection targets and identified 146, 2,494, and 4,159 potential targets of cordycepin, doxorubicin, and glioblastoma, respectively.

A total of 4,159 glioblastoma-related targets were obtained from the GeneCards database using “glioblastoma” as the keyword. The targets of cordycepin, doxorubicin, and glioblastoma were imported into a Venn diagram, and we obtained 71 intersection targets (Fig. 3).

### Biological process enrichment and pathway analysis

**Figure 4:**
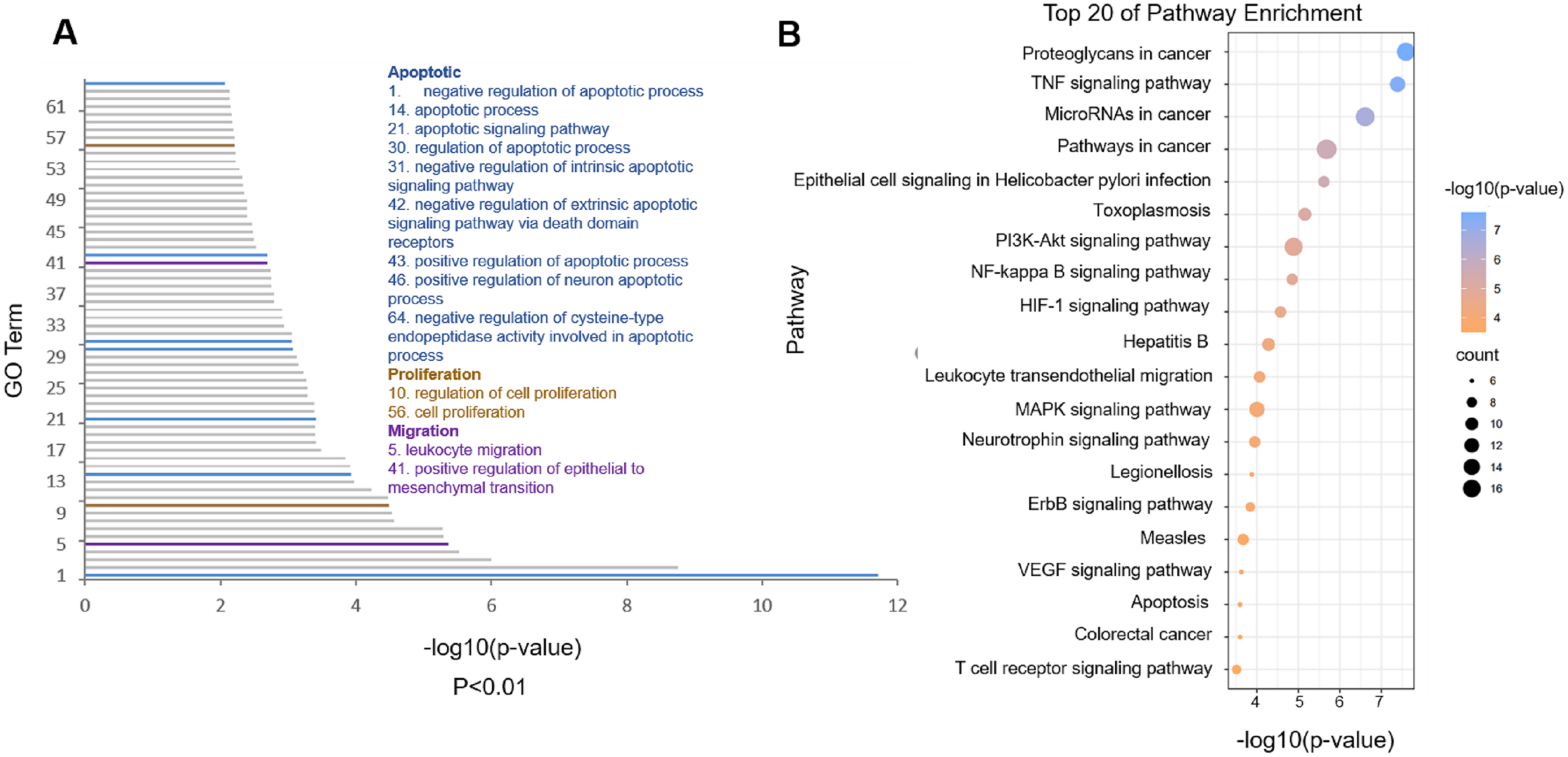
Functional analysis of the common targets. (A) Gene Ontology (GO) biological process of predicted targets (*p* < 0.01). The blue GO terms are associated with apoptosis; the light brown terms are associated with proliferation; and the purple terms are associated with migration. (B) Kyoto Encyclopedia of Genes and Genomes analysis of cordycepin combined with doxorubicin in the treatment of glioblastoma (top 20 of pathway enrichment). The bubble color is related to the *p* value, and the bubble size is related to the number of genes enriched in this pathway.

The potential targets of cordycepin combined with doxorubicin to treat glioblastoma were imported into the DAVID database for KEGG pathway enrichment analysis; the top 20 pathways with the smallest *p* values were arranged according to the *p* value, from small to large, and drawn into a bubble diagram (Fig. 4B). The abscissa of the bubble graph is –log10 (*p* value) of the pathway, and the ordinate is the name of pathway. The color represents the *p* value, and a redder *p* value indicates a more significant enrichment. The bubble size indicates the number of enrichment targets in the pathway, and the larger the bubble reflects more enriched genes.

Figure 4B shows that combination therapy to treat glioblastoma involves the pathways mainly enriched in proteoglycans in cancer, the tumor necrosis factor (TNF) signaling pathway, microRNA in cancer and pathways in cancer, among which the proteoglycans in cancer pathway had the smallest *p* value (Table 2). After analysis, 35 targets were enriched in the first four pathways. We imported 35 common targets into the DAVID database for GO biological process analysis and obtained 64 GO terms with very significant differences (*p* < 0.01). Figure 4A shows GO biological process entries with *p* values <0.01; −log10 (*p* value) is on the horizontal axis, and the GO term serial number is on the vertical axis (see the attached table for detailed information of GO terms). The entries related to cell apoptosis, proliferation, and migration are marked out. The blue entries represent the biological processes related to cell apoptosis; the red ones, cell proliferation; the black ones, cell migration. The analysis of biological processes showed that cordycepin combined with doxorubicin could regulate cell apoptosis, cell proliferation, cell migration, nucleotide synthesis, and transcription by acting on multiple targets, thus leading to the apoptosis of glioblastoma and inhibition of the migration of glioblastoma.

**Table 2:** Top 4 enriched pathways identified *via* DAVID.

Pathway	*p*	Combined	Count	Genes
Proteoglycans in cancer	2.56E−08	3,065.87	14	*PRKCA, CAV1, MMP9, TLR4, PDCD4, SRC, CTNNB1, CDC42, CASP3, PLCG1, RAC1, PLCG2, MYC, TWIST1*
TNF signaling pathway	4.08E−08	3,495.66	11	*MAP3K7, CXCL1, ICAM1, CFLAR, CASP3, CCL2, MMP9, NFKB1, MAPK8, MMP3, JUNB*
MicroRNAs in cancer	2.46E−07	2,170.72	15	*PRKCA, MCL1, MMP9, EZH2, NFKB1, ZEB1, HMGA2, PDCD4, BRCA1, CASP3, PLCG1, PLCG2, DNMT1, DNMT3B, MYC*
Pathways in cancer	2.09E−06	1,168.07	16	*PRKCA, XIAP, MMP9, NFKB1, BIRC5, MMP1, CTNNB1, CDC42, HSP90B1, CASP3, PLCG1, PAX8, PLCG2, RAC1, MAPK8, MYC*

### Construction of a drug-disease-target-pathway network and PPI network

In order to further explore the mechanism of cordycepin combined with doxorubicin in the treatment of glioblastoma, we selected the first four pathways with the lowest enriched *p* value and their related targets for analysis. The red nodes in the figure represent glioblastoma, the green nodes represent pathways, the related genes are yellow targets, and the edges between nodes represent the interaction between them.

**Figure 5:**
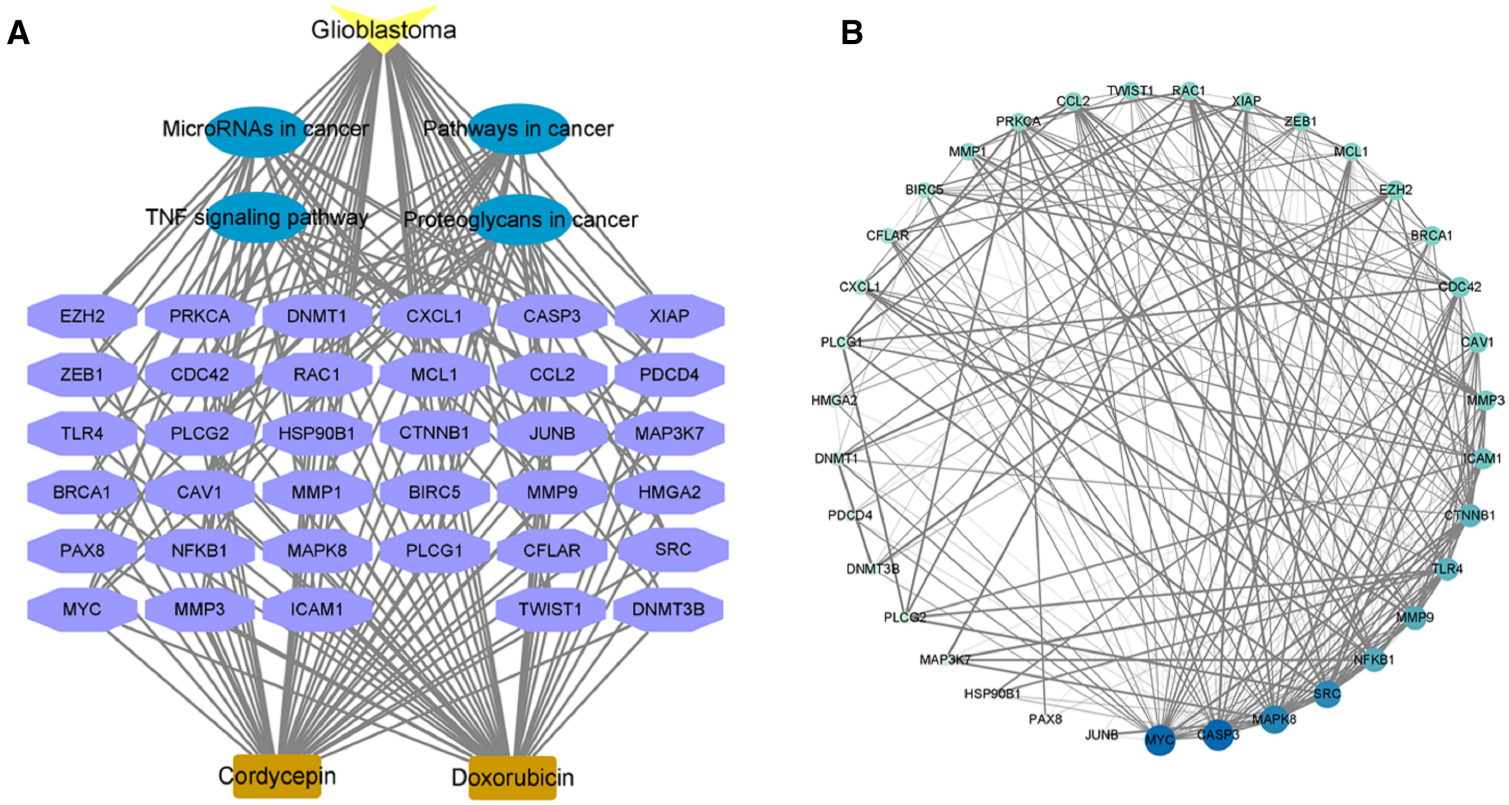
The possible targets and pathways of cordycepin combined with doxorubicin in the treatment of glioblastoma. (A) We analyzed the correlation between genes related to the first four pathways and pathways and diseases. Drug–disease–target–pathway network. The yellow color indicates glioblastoma; the blue represent the four pathways; the purple color indicates the 35 targets involved in the first four pathways; the light brown represents cordycepin and doxorubicin. (B) Protein–protein interaction network diagram construction based on the STRING database. The larger the circle and the darker the color, the larger the degree value; the thicker the line, the closer the binding between the proteins. TNF, tumor necrosis factor. Further explore the important targets of cordycepin combined with doxorubicin in the treatment of glioblastoma.

In order to further confirm the possible important targets in combination, we constructed PPI network for 35 targets enriched in the first four pathways. The 35 targets were imported into the STRING database, the species was set to *Homo sapiens*, the medium confidence was set to 0.4, and the remaining settings were kept at defaults; the data were saved as a TSV file. TSV files were imported into Cytoscape software to build the PPI network, as shown in Fig. 5B. Overall, 35 nodes interacted with each other through 226 edges. The circular nodes represent target genes, and the straight lines between nodes indicate the interaction between two linked proteins. Thicker lines represent stronger interaction relationships. The size and color depth of nodes in the figure are set by the degree value; that is, the larger the node and the darker the color, the greater the degree value of the corresponding target gene and *vice versa*. As seen in Fig. 5B, the nodes of targets such as MYC, CASP3, MAPK8, SRC, NFKB1, MMP9, TLR4, and CTNNB1 are relatively large, and the color is relatively dark, indicating a relatively large degree value. These targets may play an important role in the treatment of glioblastoma using combined cordycepin and doxorubicin.

### Validation of key targets

**Figure 6:**
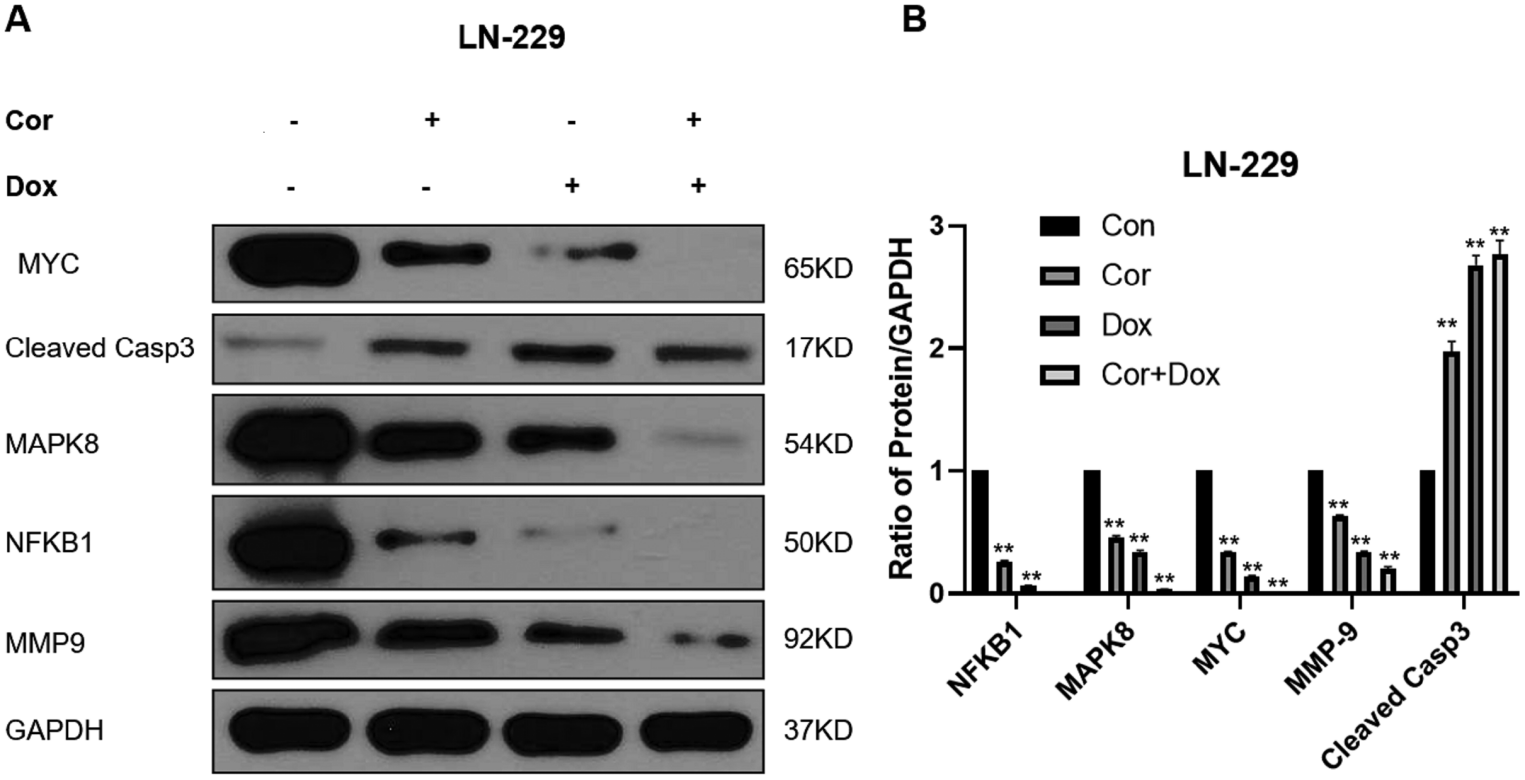
Biological validation of important targets against glioma using a combination of drugs. (A) Western blots reveal the effects of combination therapy (cordycepin and doxorubicin) on the expression levels of NFKB1, MAPK8, MYC, MMP9, and cleaved caspase 3 in LN-229 cells. (B) We used Image J to quatify the results of western bolt. GAPDH was used as a reliable internal control. Data are presented as the mean ± SD; *n* = 3; **p < 0.01 *vs* the control group (*n* = 3 represents biological repetition).

We use network pharmacology to predict the possible target of cordycepin combined with doxorubicin in the treatment of glioblastoma. Then 71 intersection genes were imported into the DAVID database for GO and KEGG analysis. GO analysis can be divided into three parts: Biological Process, Cell Component and Molecular Function. The analysis found that a total of nine items (1. negative regulation of apoptotic process; 2. apoptotic process 3. apoptotic signaling pathway 4. regulation of apoptotic process 5. negative regulation of intrinsic apoptotic signaling pathway 6. negative regulation of extrinsic apoptotic signaling pathway *via* death domain receptors 7. positive regulation of apoptotic process 8. positive regulation of neuron apoptotic process 9. negative regulation of cysteine-type endopeptidase activity involved in apoptotic process are enriched in relation to cell apoptosis), and two items (1. regulation of cell proliferation 2. cell proliferation) are related to cell proliferation. Two biological processes related to cell migration. After KEGG analysis, it was found that a total of 74 KEGG pathways were enriched, and the first top 20 pathways with *p* values from small to large were visualized using bubble charts. We analyzed the first top 4 pathways that may play an important role in the treatment of glioblastoma with cordycepin combined with doxorubicin, and were involved 35 targets. We use Cytoscape 3.8.0 to perform PPI network analysis on 35 targets. The greater the degree of the target, the more important the gene may be in the treatment of glioblastoma with cordycepin combined with doxorubicin. We detected the first few targets with a higher Degree value in the PPI network map by western blot, and explored their expression in the drug combination. Generally, the potential targets that have higher degrees value are more pharmacologically important. To explore the molecular mechanism of cordycepin combined with doxorubicin in the treatment of glioblastoma, we detected the expression levels of NFKB1, MAPK8, MYC, MMP9, and cleaved caspase 3 in LN-229 cells treated with cordycepin and doxorubicin for 48 h by western blot analysis (Fig. 6), and these genes were the targets with the highest degree values in the PPI network. NFKB1 is an important intracellular nuclear transcription factor, which is involved in inflammation and immune regulation and mainly plays a role in the nuclear factor kappa B pathway in the TNF signaling pathway (Peluso et al., 2019). MAPK8, also known as c-Jun terminal kinase 1, is an important member of the MAPK family. MAPK8 can phosphorylate and activate transcription factor activator protein-1, thereby activating the expression of a series of downstream genes, and it plays an important regulatory role in cell proliferation, cell differentiation, cell apoptosis, inflammation, and other pathological processes (Kini & Garg, 2015; Xu et al. 2018). MMP-9 is a matrix metalloproteinase that regulates cell adhesion and is associated with the occurrence, progression and local invasion of gliomas (Wu & Shen, 2021). Caspase 3 is a member of the Bcl-2 family and is the most important terminal splicing enzyme in the process of cell apoptosis; it can inhibit programmed apoptosis of tumor cells and promote proliferation of tumor cells (Humphreys, Espona-Fiedler & Longley, 2018). MYC is an oncogene encoding nuclear protein, which can be divided into C-Myc, N-Myc, and I-Myc; it is related to cell proliferation, tumorigenesis, and outcomes. We analyzed the genes related to the first top four pathways and performed PPI network analysis on them, and verified the more important targets. EMT is a relatively complex biological process, among which CASP3, MAPK8, MMP9, and NFKB1 are also EMT-related genes in the Genecards database. Onishi et al. (2011) have reported that MMP-9 maintain the rigidity of the extracellular matrix *in vitro*, and promote the structural rigidity, movement and proliferation of established glioma cell lines. What we verified is not genes that have nothing to do with cell migration. Each gene may be involved in a variety of biological processes. Among them, CASP3 and MYC are also genes related to cell apoptosis, and genes such as MAPK8 are related to cell proliferation, which also echoes the analysis of GO biological processes. The results of this study showed that, compared with the control group, the combined treatment group significantly downregulated the expression of NFKB1, MAPK8, MYC, and MMP-9 proteins; upregulated cleaved caspase 3; promoted the apoptosis of LN-229 cells; and inhibited the proliferation and migration of tumors.

## Discussion

This study proved for the first time, to our knowledge, that cordycepin combined with doxorubicin can significantly reduce the proliferation and migration of glioblastoma cells. Glioblastoma is the most common and destructive primary brain tumor. Although the current medical level is well, the prognosis of glioblastoma is still not optimistic, and glioblastoma has a tendency toward spontaneous malignant transformation. In this study, we predicted 71 potential targets of cordycepin combined with doxorubicin in the treatment of glioblastoma by network pharmacology, suggesting that cordycepin combined with doxorubicin may inhibit the growth of glioblastoma *via* multiple targets and multiple pathways.

In this study, the effects of cordycepin combined with doxorubicin on the growth, cytotoxicity, and cell proliferation of LN-229 cells were detected by observing the cell morphology, an MTT test, and a cell cloning test after the combined treatment was administered. The results showed that the combined treatment could significantly inhibit the growth, proliferation, and cell cloning of LN-229 cells. The effect of the combination therapy on the movement, invasion, and migration of LN-229 cells was detected by a cell scratch test and the expression of EMT-related genes. EMT is a process of epithelial cells transformation into mesenchymal cells under specific conditions. EMT will lose the connective structure between epithelial cells, the cytoskeleton will be remodeled, and the cell morphology will change. If the tumor cells are separated after EMT, the cells have antiapoptotic properties and can prevent the aging and apoptosis of the tumor. The cells also have increased motor capacity, which promotes cell invasion and migration. In the process of tumor EMT, tumor cells are mainly manifested by downregulated expression of epithelial markers such as E-cadherin and increased expression of mesenchymal markers such as N-cadherin and ZEB1; these changes lead to weakened intercellular adhesion and enhanced invasion and migration of tumor cells (Wang et al., 2021). Transcription factor ZEB1 promotes glioblastoma tumor progression and is negatively correlated with the survival of patients with glioblastoma (Zeng, Xiao & Guo, 2019). In this study, compared with the control group, cordycepin, doxorubicin, and combined treatment increased the E-cadherin expression and reduced the N-cadherin, Zeb1, and Twist1 protein expression as EMT protein molecular markers. These results showed that the drug combination can regulate the EMT process, and thus the glioblastoma cell invasion and migration ability; however, the specific regulatory mechanism still needs additional study. This study also tested the cell growth, proliferation and migration ability of cordycepin combined with doxorubicin on U251 and T98G cell lines through MTT assay, wound healing, colony formation assay. The results also show that the combination of drugs can effectively inhibit the growth, proliferation and migration of U251 and T98G cells, and that cordycepin combined with doxorubicin does not only have an effect on LN 229 cells, and may provide a theoretical basis for clinical treatment of glioblastoma.

To better verify the molecular mechanism of resistance to doxorubicin in glioblastoma, we used network pharmacology to predict the potential targets of treatment with cordycepin plus doxorubicin in glioblastoma, and we analyzed these targets for GO functions and on the KEGG pathway. We aimed to predict possible roles in the process of treatment pathways and detect degree values of targets. Studies have found that cordycepin combined with doxorubicin may have activity against glioblastoma through proteoglycans in cancer, the TNF signaling pathway, microRNA in cancer, pathways in cancer, and other pathways. It has been reported that many proteoglycan in the tumor environment are involved in the proliferation, adhesion, angiogenesis, and metastasis of cancer, thus affecting the progression of tumors. Rajaraman et al. (2009) showed that NFKB1 had an influence on the risk of glioma. Li et al. (2021) reported that SLC26A4-AS1 promoted the transcription activity of NPTX1 through the recruitment of NFKB1 and thus had an antiangiogenic effect on glioma cells. In a study by Xu et al. (2018), MAPK8 promoted resistance to temozolomide, accelerated cell proliferation, and inhibited the apoptosis of glioblastoma cells by activating the MAPK signaling pathway. High expression of MMP-9 is considered by the World Health Organization to be a level-III independent predictor of survival in patients with cancer (Xue et al., 2017). As Chen et al. (2019) has reported, MYC is associated with the occurrence and development of glioblastoma, and the EGFR/EGFR *vs* III enrichment MYC promoter regions will significantly promote a malignant phenotype in glioblastoma cells.

Network pharmacology is a multidisciplinary research field that interprets the development process of diseases by establishing databases, establishing networks, analyzing networks, and conducting experimental verification (Yuan et al., 2021; Zhuang, Cai & Zhang, 2021). Using omics, network pharmacology integrates systems biology, and provides a powerful tool for exploring the mechanism of action of traditional Chinese medicine or natural active substances and developing active components of traditional Chinese medicine from the perspective of biological balance. In addition, the network structure diagram of drug–target–disease can be visually presented, which can predict the target of action and signal pathway in the treatment process and then predict the potential mechanism of action—one of the effective ways to develop new drugs. Liu et al. (2021) explored the mechanism of Fangji–Huangqi decoction in the treatment of breast cancer using network pharmacology and identified 108 targets (*e.g*., interleukin-6, ALB, CASP3, vascular endothelial growth factor) and 113 pathways (*e.g*., apoptosis, TNF signaling pathway). Tu et al. (2021) studied the mechanism of action of astragalus–atractylodes in the treatment of COVID-19 and identified 19 active components and 41 targets. KEGG analysis showed that treatment of COVID-19 may involve the regulation of interleukin-17, P53, HIF, and other signaling pathways.

Cordycepin is absorbed into the peripheral blood after being taken, and then transported to the vicinity of the blood-brain barrier (BBB) through the blood circulation, and then transported into the central nervous system through a mechanism similar to adenosine transport through the blood-brain barrier, and then has an impact on the physiological functions of the brain (Jia et al., 2019). There are few reports on how cordycepin passes through the blood-brain barrier (BBB), and further research is still needed. However, Yuan et al. (2016) found that cordycepin has a neuroprotective effect on the integrity of BBB and can prevent it from brain damage caused by traumatic brain injury. First of all, most patients with glioblastoma in clinic need radiotherapy. After radiotherapy, the blood–brain barrier is destroyed, which can improve the passage of doxorubicin (Attached here is an English reference article on the treatment of glioma by doxorubicin). Secondly, most patients with glioblastoma tumors need radiotherapy. Radiotherapy opens the blood–brain barrier, which can promote the entry of chemotherapy drugs (doxorubicin) into brain tumors and improve the effect of chemotherapy. Later, in the process of glioma proliferation, this part of the barrier of the tumor area is destroyed, and part of the barrier around the tumor is also destroyed by the surgical operation. The blood-brain barrier is more likely to be breached. At present, we take glioblastoma cell lines *in vitro* as the research object. Follow-up trials will continue to explore how to improve the efficacy of doxorubicin and cordycepin in clinical applications.

## Conclusions

We proposed a new model of cytotoxic synergistic effects of cordycepin and doxorubicin on LN-229 cells that inhibit the growth, proliferation, migration, EMT, and other biological processes of LN-229 cells, and promote cell apoptosis to achieve a therapeutic effect. In addition, we used MTT experiments, wound healing and colony formation assay to verify the effect of the combination on U251 and T98G cell lines. The results show that the combination medication can also significantly inhibit the viability, migration and proliferation capabilities of U251 and T98G cells. This synergistic effect was well demonstrated in this study through network pharmacology and *in vitro* experiments. The combination of cordycepin and doxorubicin may become a new choice to treat glioblastoma, but the feasibility of the combination in clinical use still must be comprehensively evaluated in future studies.

## Supplemental Information

10.7717/peerj.12942/supp-1 Supplemental Information 1 Western blots for Figs. 2 and 6.

10.7717/peerj.12942/supp-2 Supplemental Information 2 Colony assay raw data. Raw data exported from the Image J applied for data analyses and preparation for Figs. 1C and 1F.

10.7717/peerj.12942/supp-3 Supplemental Information 3 Scratch healing raw data. Raw data exported from the Image J applied for data analyses and preparation for Figs. 2A, 2B, 2E and 2F.

10.7717/peerj.12942/supp-4 Supplemental Information 4 Raw data for Tables.

10.7717/peerj.12942/supp-5 Supplemental Information 5 Colony formation assay. Raw data of colony formation assay of three glioblastoma cell lines: LN-229, U251 and T98G. Raw data exported from the Image J applied for data analyses and preparation for the detailed investigation shown Figs. 1C and 1G.

10.7717/peerj.12942/supp-6 Supplemental Information 6 MTT assay. Raw data of MTT assay of three glioblastoma cell lines: LN-229, U251 and T98G.Raw data exported from the Microplate reader applied for data analyses and preparation for Figs. 1B and 1E.

10.7717/peerj.12942/supp-7 Supplemental Information 7 Western blot raw data. Raw data exported from the Image J applied for data analyses and preparation for the detailed investigation shown Figs. 2 and 6 for the protein.

10.7717/peerj.12942/supp-8 Supplemental Information 8 Figure of the results of repeated Western blot experiments. This is a picture of the result of repeated experiment 1 by Western blot. REPEAT1 represents the result of repeated experiment 1, “REPEAT2”stands for repeated test 2, Cor stands for Cordycepin, Dox stands for Doxorubicin, Cor Dox stands for Cordycepin combined with Doxorubicin and Con stands for control group.

10.7717/peerj.12942/supp-9 Supplemental Information 9 Wound healing assay. Raw data of wound healing assay of three glioblastoma cell lines: LN-229, U251 and T98G. Raw data exported from the Image J applied for data analyses and preparation for Figs. 2A, 2B, 2E and 2F.

10.7717/peerj.12942/supp-10 Supplemental Information 10 WB raw data.

10.7717/peerj.12942/supp-11 Supplemental Information 11 Repeat Western blot.

10.7717/peerj.12942/supp-12 Supplemental Information 12 Repeated Western blot for Fig. 6.

10.7717/peerj.12942/supp-13 Supplemental Information 13 Concentration screening of combined medication. Pre-experimental data for screening the concentration of cordycepin and doxorubicin in combination.

10.7717/peerj.12942/supp-14 Supplemental Information 14 Appendix 1. Information about related target genes predicted during the treatment of glioma by cordycepin combined with doxorubicin.

10.7717/peerj.12942/supp-15 Supplemental Information 15 Appendix 2. Issues related to blood-brain barrier.

## Abbreviations

Cor: cordycepin
Dox: doxorubicin
BS: Tris-buffered saline
EMT: Epithelial-Mesenchymal Transition
ACHN: Adjunctive cells of human renal cell adenocarcinoma
HPLC: High Performance Liquid Chromatography
OD: Optical Density

## Additional Information and Declarations

### Data Availability

The following information was supplied regarding data availability: The raw measurements are available in the Supplemental Files.

## References

[ref-44] Bandak M, Jørgensen N, Juul A, Lauritsen J, Oturai PS, Mortensen J, Hojman P, Helge JW, Daugaard G (2017). Leydig cell dysfunction, systemic inflammation and metabolic syndrome in long-term testicular cancer survivors. European Journal of Cancer.

[ref-1] Bremnes RM, Veve R, Gabrielson E, Hirsch FR, Baron A, Bemis L, Gemmill RM, Drabkin HA, Franklin WA (2002). High-throughput tissue microarray analysis used to evaluate biology and prognostic significance of the E-cadherin pathway in non-small-cell lung cancer. Journal of Clinical Oncology.

[ref-2] Chaicharoenaudomrung N, Jaroonwitchawan T, Noisa P (2017). Cordycepin induces apoptotic cell death of human brain cancer through the modulation of autophagy. Toxicology In Vitro.

[ref-45] Chen YF, Wang S, Li B (2012). MTT assay for cytotoxic drugs on experimental study of L2 Cells. Progress in Modern Biomedicine.

[ref-3] Chen X, Yang F, Zhang T, Wang W, Xi W, Li Y, Zhang D, Huo Y, Zhang J, Yang A, Wang T (2019). MiR-9 promotes tumorigenesis and angiogenesis and is activated by MYC and OCT4 in human glioma. Journal of Experimental & Clinical Cancer Research.

[ref-4] Chen YC, Chen YH, Pan BS, Chang MM, Huang BM (2017). Functional study of Cordyceps sinensis and cordycepin in male reproduction: a review. Journal of Food and Drug Analysis.

[ref-5] Deng H, Mo W, Luo Y (2011). The application of adriamycin controlled sustained release chemotherapy and postoperative local chemotherapy in the treatment of glioma. Chongqing Medicine.

[ref-6] Fan H, Zhu YJ, Zhao YF (2019). Effect of cordycepin combined with gemcitabine on apoptosis of breast cancer cells and its mechanism. Chinese Journal of Gerontology.

[ref-7] Franken N, Rodermond H, Stap J, Haveman J, van Bree C (2006). Clonogenic assay of cells In Vitro. Nature Protocols.

[ref-8] Gao S, Ma J, Liu J, Zhao G (2021). Preliminary study on antibacterial activity and mechanism of cordycepin. Biotechnology Bulletin.

[ref-9] Guo Z, Chen W, Dai G, Huang Y (2019). Cordycepin suppresses the migration and invasion of human liver cancer cells by downregulating the expression of CXCR4. International Journal of Molecular Medicine.

[ref-10] Han Y, Sun Y, Zhang Y, Xia Q (2020). High DPP4 expression predicts poor prognosis in patients with low-grade glioma. Molecular Biology Reports.

[ref-11] Humphreys L, Espona-Fiedler M, Longley DB (2018). FLIP as a therapeutic target in cancer. FEBS Journal.

[ref-12] Jang KJ, Kwon GS, Jeong JW, Kim CH, Yoon HM, Kim GY, Shim JH, Moon SK, Kim WJ, Choi YH (2015). Cordyceptin induces apoptosis through repressing hTERT expression and inducing extranuclear export of hTERT. Journal of Bioscience and Bioengineering.

[ref-13] Jia Y, Haoran L, Tangxin G, Bai L, Hongkun B, Jing D (2019). The pharmacological basis of cordycepin for brain protection and mood stabilization through adenosine receptors.

[ref-14] Jing H, Zhang H, Qin D (2021). Progressions of imaging studies on recurrence and treatment of brain glioma. Chinese Medicine and Clinics.

[ref-15] Kini SG, Garg V (2015). JNK pathway signaling: a novel and smarter therapeutic targets for various biological diseases. Future Medicinal Chemistry.

[ref-16] Li H, Yan R, Chen W, Ding X, Liu J, Chen G, Zhao Q, Tang Y, Lv S, Liu S, Yu Y (2021). Long non coding RNA SLC26A4-AS1 exerts antiangiogenic effects in human glioma by upregulating NPTX1 via NFKB1 transcriptional factor. The FEBS Journal.

[ref-17] Liu Y, Fan Y, Yu H, Guo Y, Yu B, Wang C, Pei X (2021). Study on the mechanism of Fangjihuangqi decoction in treating breast cancer based on network pharmacology. Journal of Modern Oncology.

[ref-18] Li J, Chen W, Huang W (2019). Research progress on anti-cancer activity of cordycepin in Cordyceps militaris. Pharmacy Today.

[ref-19] Liao Y, Ling J, Zhang G, Liu F, Tao S, Han Z, Chen S, Chen Z, Le H (2015). Cordycepin induces cell cycle arrest and apoptosis by inducing DNA damage and up-regulation of p53 in leukemia cells. Cell Cycle.

[ref-20] Louis DN, Perry A, Wesseling P, Brat DJ, Cree IA, Figarella-Branger D, Hawkins C, Ng HK, Pfister SM, Reifenberger G, Soffietti R, von Deimling A, Ellison DW (2021). The 2021 WHO Classification of Tumors of the Central Nervous System: a summary. Neuro-Oncology.

[ref-21] Onishi M, Ichikawa T, Kurozumi K, Date I (2011). Angiogenesis and invasion in glioma. Brain Tumor Pathology.

[ref-22] Park JG, Son YJ, Lee TH, Baek NJ, Yoon DH, Kim TW, Aravinthan A, Hong S, Kim JH, Sung GH, Cho JY (2017). Anticancer efficacy of Cordyceps militaris ethanol extract in a xenografted leukemia model. Evidence-Based Complementary and Alternative Medicine.

[ref-23] Peluso I, Yarla NS, Ambra R, Pastore G, Perry G (2019). MAPK signalling pathway in cancers: olive products as cancer preventive and therapeutic agents. Seminars in Cancer Biology.

[ref-24] Rajaraman P, Brenner AV, Butler MA, Wang SS, Pfeiffer RM, Ruder AM, Linet MS, Yeager M, Wang Z, Orr N, Fine HA, Kwon D, Thomas G, Rothman N, Inskip PD, Chanock SJ (2009). Common variation in genes related to innate immunity and risk of adult glioma. Cancer Epidemiology Biomarkers & Prevention.

[ref-25] Syam S, Bustamam A, Abdullah R, Sukari MA, Hashim NM, Ghaderian M, Rahmani M, Mohan S, Abdelwahab SI, Ali HM (2014). β-Mangostin induces p53-dependent G2/M cell cycle arrest and apoptosis through ROS mediated mitochondrial pathway and NfkB suppression in MCF-7 cells. Journal of Functional Foods.

[ref-26] Tania M, Shawon J, Saif K, Kiefer R, Khorram MS, Halim MA, Khan MA (2019). Cordycepin downregulates Cdk-2 to interfere with cell cycle and increases apoptosis by generating ROS in cervical cancer cells: In Vitro and In Silico study. Current Cancer Drug Targets.

[ref-27] Tu J, Wang B, Xia W (2020). Research progress on biological active components and pharmacological action of Cordyceps militaris. Edible Fungi.

[ref-28] Tu W, Zhe J, Li M, Chen M, Yang Z, Gan H (2021). Study on the mechanism of astragalus membranaceus-atractylodes macrocephalae in treatment of COVID-19 based on network pharmacology. Fujian Journal of Traditional Chinese Medicine.

[ref-29] Wang S, Li B (2013). MTT assay for cytotoxic drugs on experimental study of L2 cells. Progress in Modern Biomedicine.

[ref-30] Wang W, Hao Y, Zhang A, Yang W, Wei W, Wang G, Jia Z (2021). miR-19a/b promote EMT and proliferation in glioma cells via SEPT7-AKT-NF-κB pathway. Molecular Therapy - Oncolytics.

[ref-31] Wu J, Shen H (2021). Correlation between the quantitative parameters of MR diffusion tensor imaging and the expression of VEGF and MMP-9 in brain gliomas. Imaging Science and Photochemistry.

[ref-32] Xu P, Zhang G, Hou S, Sha LG (2018). MAPK8 mediates resistance to temozolomide and apoptosis of glioblastoma cells through MAPK signaling pathway. Biomedicine & Pharmacotherapy.

[ref-33] Xue Q, Cao L, Chen XY, Zhao J, Gao L, Li SZ, Fei Z (2017). High expression of MMP9 in glioma affects cell proliferation and is associated with patient survival rates. Oncology Letters.

[ref-34] Yang Y, Wang J, Shi F, Shan A, Xu S, Lv W (2021). BDKRB2 is a novel EMT-related biomarker and predicts poor survival in glioma. Aging.

[ref-35] Yuan J (2017). Effects of cordycepin on blood brain barrier damage in rats with traumatic brain injury.

[ref-36] Yuan J, Wang A, He Y, Si Z, Xu S, Zhang S, Wang K, Wang D, Liu Y (2016). Cordycepin attenuates traumatic brain injury-induced impairments of blood-brain barrier integrity in rats. Brain Research Bulletin.

[ref-37] Yuan T, Cui L, Wang Y, Li Y, Liu Y (2021). Latest development of TCM network pharmacology. Acta Chinese Medicine and Pharmacology.

[ref-38] Zeng Y, Xiao H, Guo Q (2019). Advances in epithelial-mesenchymal transition of glioblastoma. Chinese Journal of Diagnostic Pathology.

[ref-39] Zhang M, Niu F, Zhao D (2021). Application of combined detection of CA199, NSE, CA153 and CEA in diagnosis and prognosis analysis of brain glioma. International Journal of Laboratory Medicine.

[ref-40] Zhao W, Zuo N, Ge C, Cai H (2018). Promoting effect of HPV16 E6 on ZEB1-mediated EMT in cervical cancer cells. Medical Journal of Wuhan University.

[ref-41] Zhang M, Chen Z, Huo Y, Wang S, Li Z, Wang X, Sheng J, Gui M, Ma X (2021). Mechanism of cordycepin combined with adriamycin on of renal carcinoma ACHN cell line. Journal of Modern Oncology.

[ref-42] Zhou Q, Zhang Z, Song L, Huang C, Cheng Q, Bi S, Hu X, Yu R (2018). *Cordyceps militaris* fraction inhibits the invasion and metastasis of lung cancer cells through the protein kinase B/glycogen synthase kinase 3β/β-catenin signaling pathway. Oncology Letters.

[ref-43] Zhuang Y, Cai B, Zhang Z (2021). Application progress of network pharmacology in traditional Chinese medicine research. Journal of Nanjing University of Chinese Medicine.

