# Supplementary material for: Exploring the mechanism of cordycepin combined with doxorubicin in treating glioblastoma based on network pharmacology and biological verification
Source: PeerJ. 2022 Feb 15;10:e12942. doi: 10.7717/peerj.12942 (PMC8855715; doi:10.7717/peerj.12942)
Supplement: Supplemental Information 1 [file peerj-10-12942-s001.pdf]

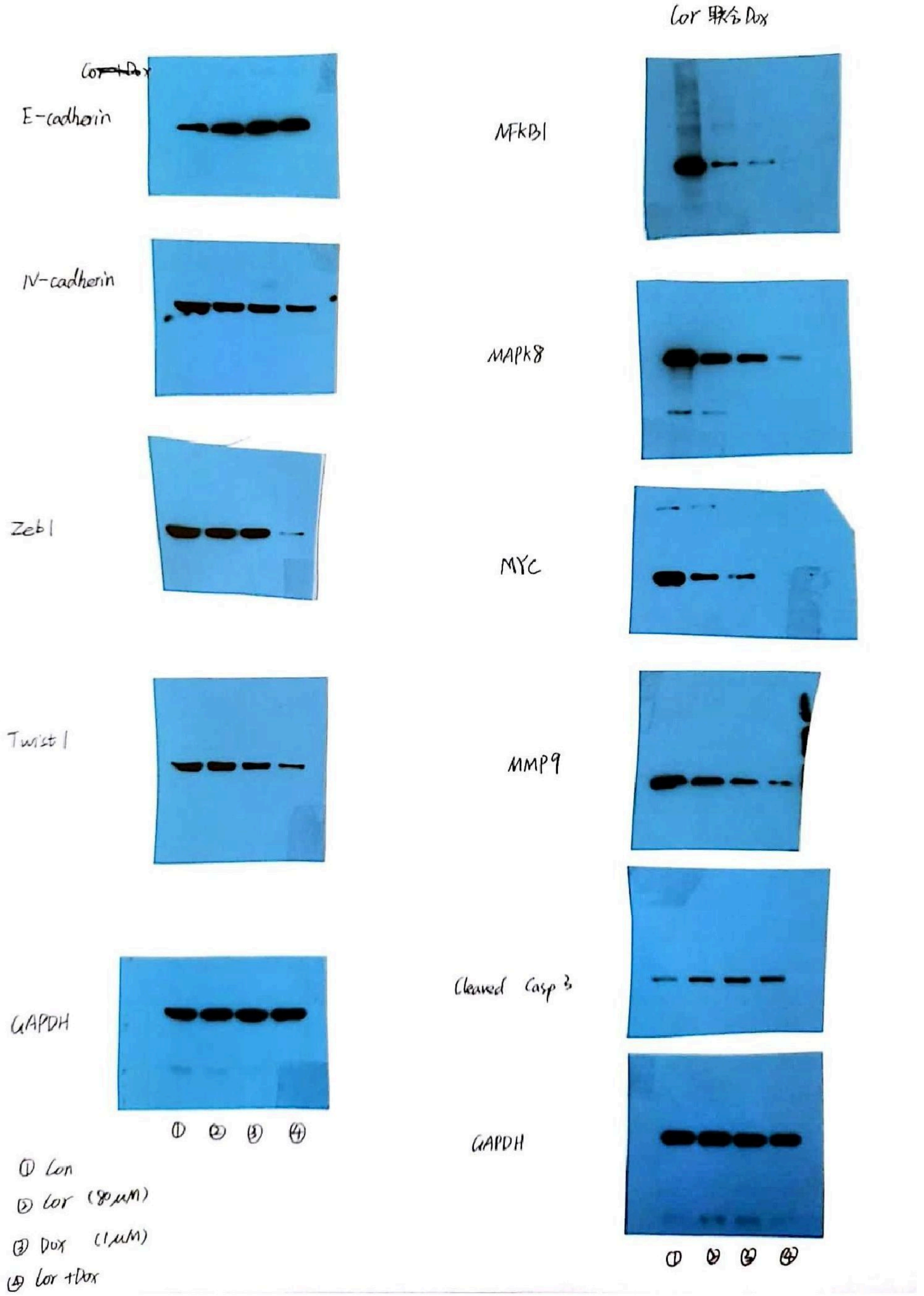

来自 扫描全能王免费版
手机上的文档、证件扫描识别利器
扫描快速下载到智能设备
