# Supplementary material for: Exploring the mechanism of cordycepin combined with doxorubicin in treating glioblastoma based on network pharmacology and biological verification
Source: PeerJ. 2022 Feb 15;10:e12942. doi: 10.7717/peerj.12942 (PMC8855715; doi:10.7717/peerj.12942)
Supplement: Supplemental Information 8 — This is a picture of the result of repeated experiment 1 by Western blot. REPEAT1 represents the result of repeated experiment 1, “REPEAT2”stands for repeated test 2, Cor stands for Cordycepin, Dox stands for Doxorubicin, Cor Dox stands for Cordycepin combined with Doxorubicin and Con stands for control group. [file peerj-10-12942-s008.pdf]

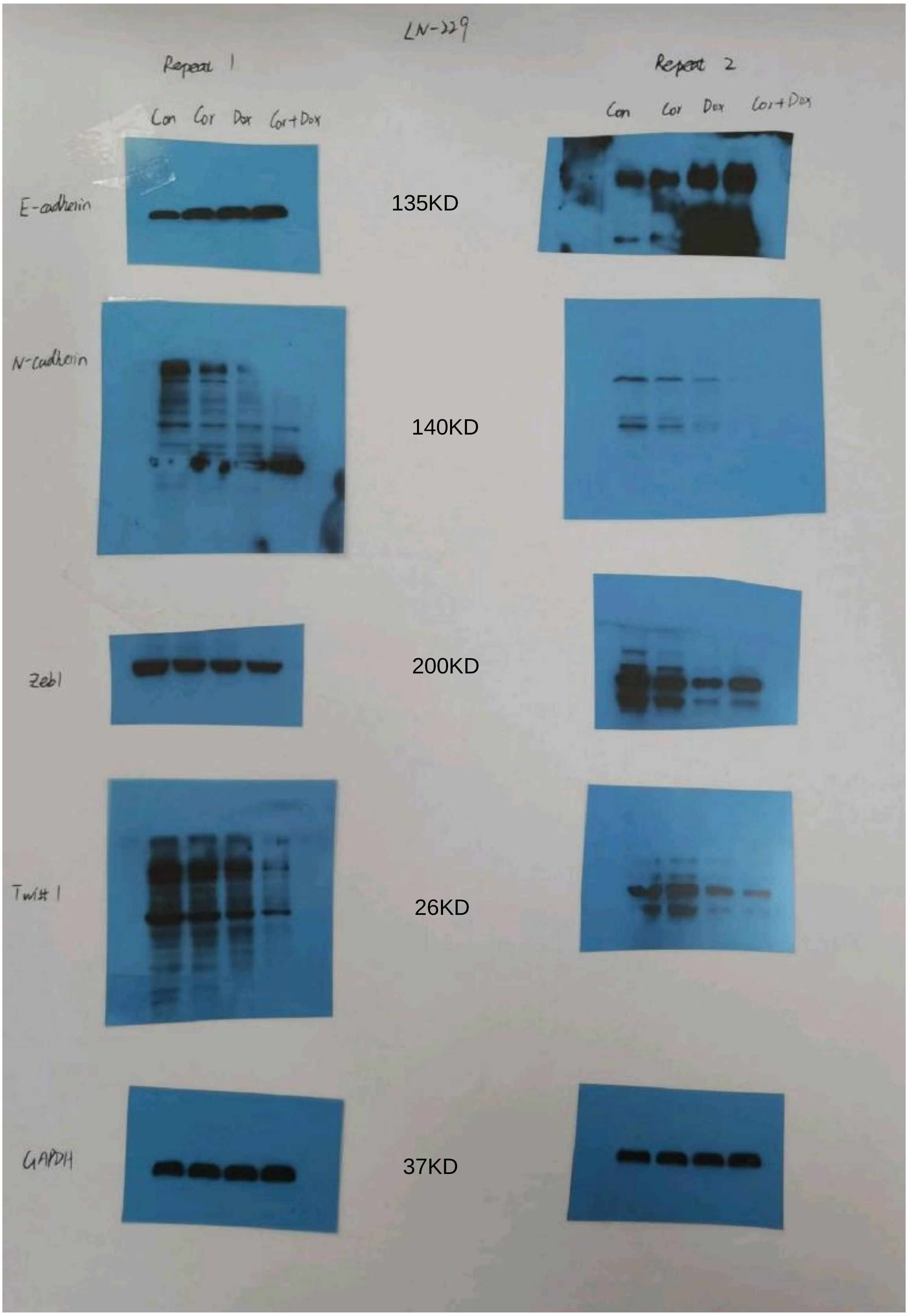
LN-229
Repeat 1
Repeat 2
Con Cor Dox Cor+Dox
Con Cor Dox Cor+Dox
E-cadherin
135KD
N-cadherin
140KD
Zeb1
200KD
Twist 1
26KD
GAPDH
37KD
