# Supplementary figures and images for: Exploring the mechanism of cordycepin combined with doxorubicin in treating glioblastoma based on network pharmacology and biological verification
Source: PeerJ. 2022 Feb 15;10:e12942. doi: 10.7717/peerj.12942 (PMC8855715; doi:10.7717/peerj.12942)

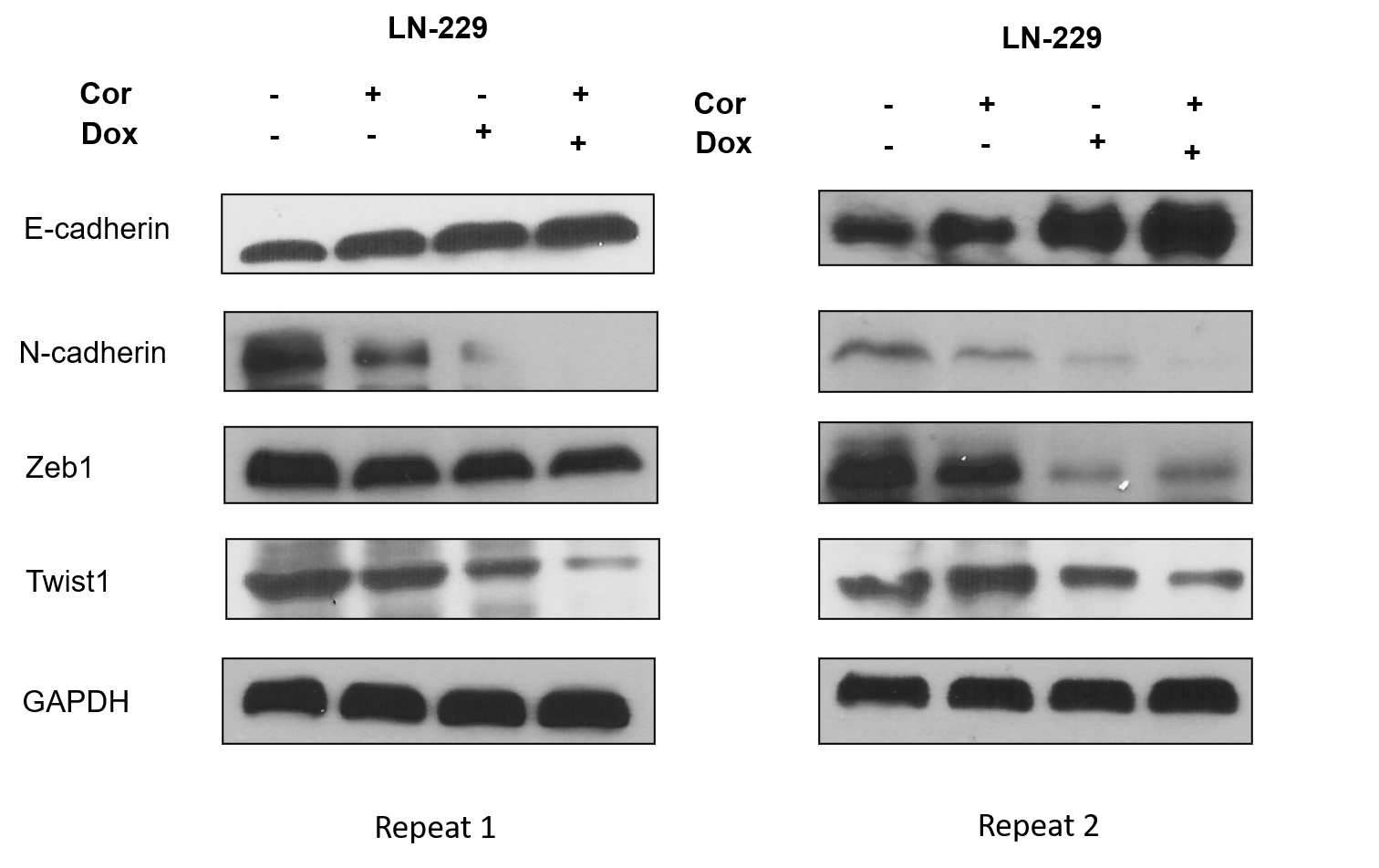

Supplement: Supplemental Information 11 [file peerj-10-12942-s011.png]

LN-229

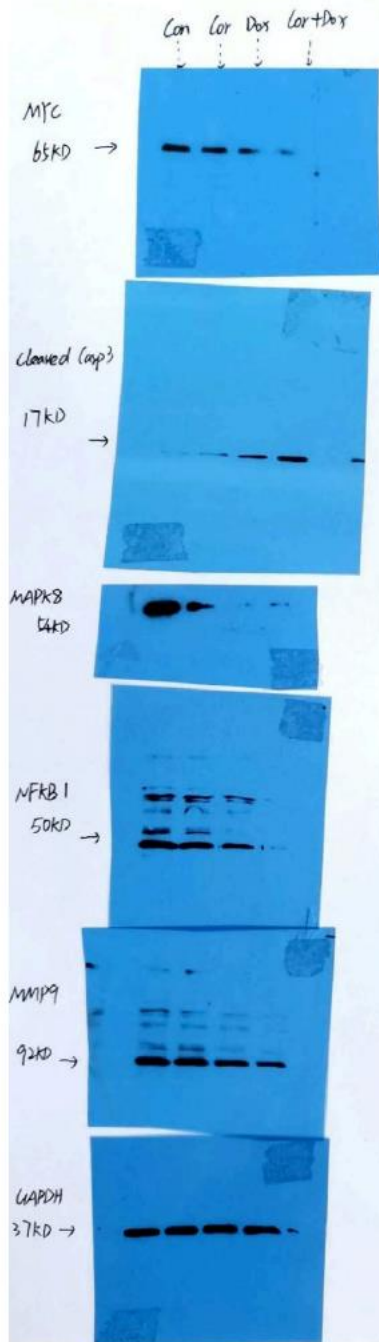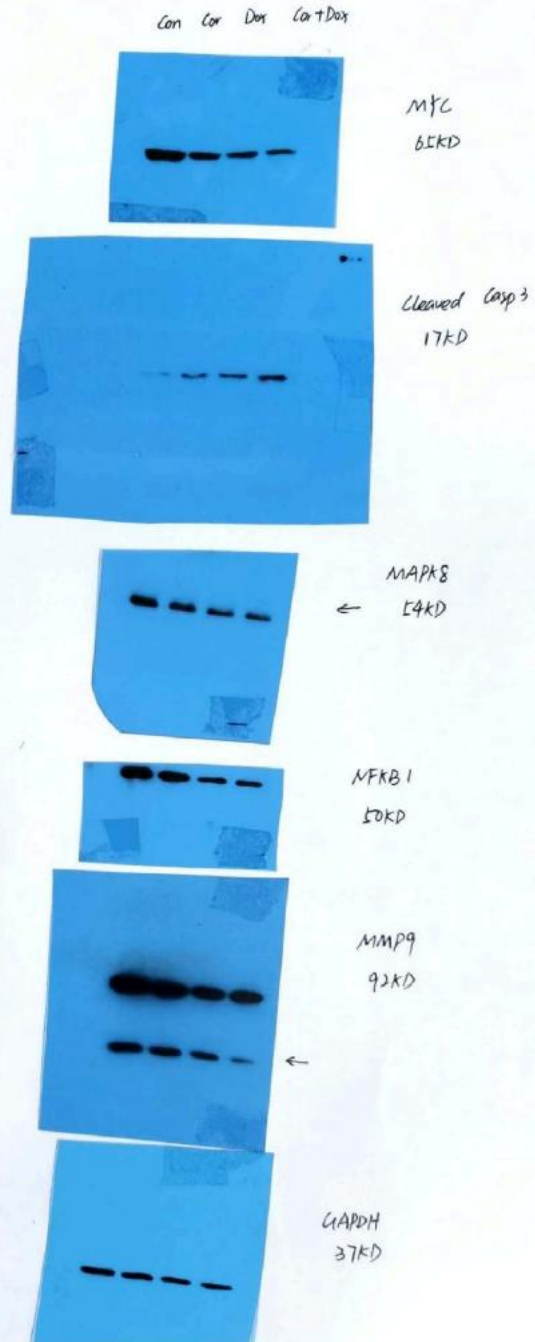

Supplement: Supplemental Information 12 [file peerj-10-12942-s012.pdf]
