# Supplementary material for: Exploring the mechanism of cordycepin combined with doxorubicin in treating glioblastoma based on network pharmacology and biological verification
Source: PeerJ. 2022 Feb 15;10:e12942. doi: 10.7717/peerj.12942 (PMC8855715; doi:10.7717/peerj.12942)
Supplement: Supplemental Information 15 — Issues related to blood-brain barrier. [file peerj-10-12942-s015.docx]

The authors have proven that the combination of doxorubicin with cordycepin show anti-proliferative activity. As the authors clearly state int their introduction, doxorubicin is an effective anti-cancer drug with cytotoxicity in glioma, but its effectiveness in treating glioblastoma is constrained by insufficient penetration across the blood–brain barrier (BBB). How would the authors suggest to go around this obstacle? On the same subject, cordycepin BBB penetrance is still unknown. What is the feasibility of this treatment combination to reach the clinic?

**1. The lipid-soluble doxorubicin liposomes can easily penetrate the lipid bilayer and improve the permeability of the blood-brain barrier, or nanoparticles can be used as carriers to penetrate the blood-brain barrier**

1. 1) Su et al. developed the BBB stealth iron oxide (Fe_3_O_4_) nanocomposite nanoparticles (NPs) as a safe nano carrier for Glioblastoma multiforme therapy[1].
2. 2) Eunseok et al. researched a novel quaternary ammonium salt-cyclodextrin (QACD) Nanoparticle as a drug delivery carrier that enables doxorubicin (DOX), a hydrophobic anticancer drug, to cross the blood-brain barrier (BBB)[2].
3. I Steiniger SC et al. have showned that doxorubixin binds to polylactide-coated nanoparticles that cross the complete blood-brain barrier to reach therapeutic concentrations in the brain. In addition, the combination of Doxorubicin with nanoparticles can reduce the systemic toxicity.
4. Coated PBCA nanoparticles have great potential for delivering drugs to central nervous system[4].
5. Pinzón-Daza, M.L. et al. have turned drugs with poor permeability (e.g. amycin) into drugs that can cross the blood-brain barrier (using liposomes)[5].
6. Because of the existence of the blood-brain barrier (BBB) , the diffusion of endogenous molecules is strictly regulated by the blood-brain barrier (BBB) . Glutathione Polyethylene Glycol Liposomal Doxorubicin (2B3-101) is a new therapeutic option for patients with brain cancer. It is based on a commercially available Polyethylene glycol liposome, DoxilH Caelyxh, and has an additional Glutathione coating that safely enhances drug delivery through the blood-brain barrier[6].

**2.** **Most Glioblastoma multiforme patients need radiation therapy. After radiotherapy, the blood-brain barrier is destroyed, which can improve the passability of doxorubicin.**

1）. Radiotherapy with a dose of 20-30 Gy and a fraction of 2 gy can be used to increase BBB permeability. Brain metastases must be investigated as a possible combination of chemoradiotherapy, which is a way of reducing the burden on the tumor and has the advantage of destroying the BBB for more effective chemotherapy[7].

2). Effects of ionizing radiation on blood-brain barrier in vitro model γ-ray irradiation induced increase in BBB permeability of [14C ]-sucrose, which could be detected 72 hours after exposure to doses up to 4 gy[8].

3）The blood-brain barrier permeability of GEFITINIB increased with the dose of WBRT[9].

1. In the process of glioblastoma proliferation, this part of the barrier of the tumor area is destroyed, plus the part of the barrier around the tumor is also destroyed by surgery. The blood-brain barrier is easier to breach.
2. Tumor-derived EV can destroy the intact BBB *in vivo*, and by using the most advanced animal models in vitro and in Vivo in the blood-brain barrier, we have established the basis of the mechanism of cross-cellular interaction in this process. In addition, high-spatial-temporal resolution microscopy showed that the endothelial endocytosis pathway is involved in this transcellular transport. We further identify and describe the mechanism by which NEOPLASTIC EV bypasses the blood-brain barrier by reducing the low physiological rate of cell transport in the blood-brain barrier[10].

2）Superselective intra-arterial mannitol infusion, focused ultrasound, laser interstitial thermotherapy and non-thermal irreversible electroporation (NTIRE). These techniques can lead to transient blood-brain barrier and blood-brain barrier disruption, and allow for more effective local drug delivery potential. Animal Studies and preliminary clinical trials show promise. Superselective intra-arterial mannitol infusion, focused ultrasound, laser interstitial thermotherapy and non-thermal irreversible electroporation (NTIRE). These techniques can lead to transient blood-brain barrier and blood-brain barrier disruption, and allow for more effective local drug delivery potential. Animal Studies and preliminary clinical trials show promise[11].

4. Pulsed ultrasound destroys the blood brain barrier

1）Liu et al. used focused ultrasound to enhance 1,3-bis (2-chloroethyl)-1-nitrosourea (BCNU) delivery of Glioblastoma multiforme in tumor-inducing rats and to determine whether this approach would improve therapeutic outcomes[12]. This study demonstrates a method to increase the delivery volume of local chemotherapy drugs for brain tumor therapy, and strongly supports the clinical feasibility of this treatment.

2）Magnetic Resonance image-guided focused ultrasound (MRgFUS) has been used to achieve a brief BBB opening without damaging tissue. ADRIAMYCIN IS CYTOTOXIC to all three cell lines and is known to have poor BBB permeability. The highest number was measured in the brain stem of NSG mice in the group receiving MRgFUS and microbubbles (431.5 ng/g) [13]. This was significantly higher than that of the untreated mice (7.6 Ng/g) . After the opening of the MRgFUS to the BBB, the histological and functional integrity of the brainstem was preserved, and the drug delivery to this area was significantly enhanced, promising treatment for specific brain stem diseases.

3）ULTRASOUND-MEDIATED BBB disruption may significantly enhance the antitumor efficacy of doxorubicin liposomes[14].

**Reference:**

(1) Su CH, Tsai CY, Tomanek B, Chen WY, Cheng FY. Evaluation of blood-brain barrier-stealth nanocomposites for in situ glioblastoma theranostics applications. Nano*scale. Ap*r 21 2016;8(15):7866-70. doi:10.1039/c6nr00280c

(2) Eun Seok. Gil,Jianshu Li,Huining Xiao, et al Quaternary Ammonium -Cyclodextrin Nanoparticles for Enhancing Doxorubicin Permeability across the In Vitro Blood-Brain Barrier Biomacromolecules 2009, 10, 505–516.

1. I Steiniger SC, Kreuter J, Khalansky AS, et al. Chemotherapy of glioblastoma in rats using doxorubicin-loaded nanoparticles. nt *J Cancer. Ma*y 1 2004;109(5):759-67. doi:10.1002/ijc.20048
2. Wohlfart. S, Khalansky AS, Gelperina S, Begley D, Kreuter J. Kinetics of transport of doxorubicin bound to nanoparticles across the blood-brain barrier. J Control Release. Aug 25 2011;154(1):103-7. doi:10.1016/j.jconrel.2011.05.010
3. Pinzón‐Daza, M. L., et al. "The association of statins plus LDL receptor‐targeted liposome‐encapsulated doxorubicin increases in vitro drug delivery across blood–brain barrier cells." British journal of pharmacology 167.7 (2012): 1431-1447.
4. Gaillard, Pieter J., et al. "Pharmacokinetics, brain delivery, and efficacy in brain tumor-bearing mice of glutathione pegylated liposomal doxorubicin (2B3-101)." PloS one 9.1 (2014): e82331.
5. Marco van Vulpen, HBK, Martin J B Taphoorn, . Changes in blood-brain barrier permeability induced by radiotherapy: implications for timing of chemotherapy? (Review). col Rep. 2002.
6. Fauquette W, Amourette C, Dehouck M-P, Diserbo M. Radiation-induced blood–brain barrier damages: An in vitro study. *Brain research*. 2012;1433:114-126.
7. Zeng YD, Liao H, Qin T, Zhang L, Wei WD, Liang JZ, Xu F, Dinglin XX, Ma SX, Chen LK. Blood-brain barrier permeability of gefitinib in patients with brain metastases from non-small-cell lung cancer before and during whole brain radiation therapy. Oncotarget. 2015 Apr 10;6(10):8366-76. doi: 10.18632/oncotarget.3187.
8. Morad G, Carman CV, Hagedorn EJ, Perlin JR, Zon LI, Mustafaoglu N, Park TE, Ingber DE, Daisy CC, Moses MA. Tumor-Derived Extracellular Vesicles Breach the Intact Blood-Brain Barrier via Transcytosis. ACS Nano. 2019 Dec 24;13(12):13853-13865. doi: 10.1021/acsnano.9b04397. Epub 2019 Sep 10.
9. Rodriguez A, Tatter SB, Debinski W. Neurosurgical Techniques for Disruption of the Blood-Brain Barrier for Glioblastoma Treatment. Pharmaceutics. Aug 3 2015;7(3):175-87. doi:10.3390/pharmaceutics7030175.
10. Liu HL, Hua MY, Chen PY, Chu PC, Pan CH, Yang HW, Huang CY, Wang JJ, Yen TC, Wei KC. Blood-brain barrier disruption with focused ultrasound enhances delivery of chemotherapeutic drugs for glioblastoma treatment. Radiology. 2010 May;255(2):415-25. doi: 10.1148/radiol.10090699. PMID: 20413754.
11. Alli S, Figueiredo CA, Golbourn B, et al. Brainstem blood brain barrier disruption using focused ultrasound: A demonstration of feasibility and enhanced doxorubicin delivery. J Control Release. Jul 10 2018;281:29-41. doi:10.1016/j.jconrel.2018.05.005.
12. Treat LH, McDannold N, Zhang Y, Vykhodtseva N, Hynynen K. Improved anti-tumor effect of liposomal doxorubicin after targeted blood-brain barrier disruption by MRI-guided focused ultrasound in rat glioma. Ultrasound Med Biol. Oct 2012;38(10):1716-25. doi:10.1016/j.ultrasmedbio.2012.04.015.
